# Lorentz-regularized interpretable VAE for multi-scale single-cell transcriptomic and epigenomic embeddings

**DOI:** 10.3389/fgene.2025.1713727

**Published:** 2026-01-05

**Authors:** Zeyu Fu, Jiawei Fu, Chunlin Chen, Keyang Zhang, Song Wang

**Affiliations:** 1 State Key Laboratory of Trauma and Chemical Poisoning, Institute of Combined Injury, Chongqing Engineering Research Center for Nanomedicine, College of Preventive Medicine, Army Medical University, Chongqing, China; 2 Department of Orthopedics, Xinqiao Hospital, Army Medical University, Chongqing, China; 3 Department of Rehabilitation Medicine, The First Affiliated Hospital, Sun Yat-sen University, Guangzhou, China; 4 School of Medicine, Sun Yat-sen University, Shenzhen, China

**Keywords:** single-cell multi-omics, dual-pathway c, hyperbolic geometry, information bottleneck, manifold learning, interpretable representation

## Abstract

**Background:**

Single-cell multi-omics technologies capture cellular heterogeneity at unprecedented resolution, yet dimensionality reduction methods face a fundamental local–global trade-off: approaches optimized for local neighborhood preservation distort global topology, while those emphasizing global coherence obscure fine-grained cell states.

**Results:**

We introduce the Lorentz-regularized variational autoencoder (LiVAE), a dual-pathway architecture that applies hyperbolic geometry as soft regularization over standard Euclidean latent spaces. A primary encoding pathway preserves local transcriptional details for high-fidelity reconstruction, while an information bottleneck (BN) pathway extracts global hierarchical structure by filtering technical noise. Lorentzian distance constraints enforce geometric consistency between pathways in hyperbolic space, enabling LiVAE to balance local fidelity with global coherence without requiring specialized batch-correction procedures. Systematic benchmarking across 135 datasets against 21 baseline methods demonstrated that LiVAE achieves superior global topology preservation (distance correlation gains: 0.209–0.436), richer latent geometry (manifold dimensionality: 0.123–0.467; participation ratio: 0.149–0.761), and enhanced robustness (noise resilience: 0.184–0.712) while maintaining competitive local fidelity. The overall embedding quality improved by 0.051–0.284 across uniform manifold approximation and projection (UMAP) and t-distributed stochastic neighbor embedding (t-SNE) visualizations. Component-wise interpretability analysis on a *Dapp1* perturbation dataset revealed biologically meaningful latent axes.

**Conclusion:**

LiVAE provides a robust, general-purpose framework for single-cell representation learning that resolves the local–global trade-off through geometric regularization. By maintaining Euclidean latent spaces while leveraging hyperbolic priors, LiVAE enables improved developmental trajectory inference and mechanistic biological discovery without sacrificing compatibility with existing computational ecosystems.

## 1 Introduction

Cellular development unfolds through hierarchical differentiation programs where stem cells progressively commit to specialized fates (Trapnell et al., 2014). Single-cell multi-omics technologies now capture these hierarchies at an unprecedented resolution (Stuart et al., 2019); however, representing tree-like developmental structures computationally remains an open challenge (Luecken et al., 2022). Datasets routinely contain $10^{5}$ – $10^{6}$ cells spanning dozens of cell types across tissues (Hao et al., 2021), demanding representations that preserve both fine-grained cell states and global developmental trajectories.

This challenge manifests as a fundamental local–global trade-off: representations must capture fine-grained local neighborhoods for rare cell-type detection (Heiser and Lau, 2020; Kiselev et al., 2019) while maintaining global topology for developmental trajectory inference (Cao et al., 2019; Saelens et al., 2019). Despite advances in deep learning (Hu et al., 2021; Yuan and Kelley, 2022) and foundation models (Cui et al., 2024), most methods excel at one scale at the expense of the other. The tension reflects a geometric limitation of Euclidean spaces: methods optimized for local structure, such as t-distributed stochastic neighbor embedding (t-SNE) (Kobak and Berens, 2019) and uniform manifold approximation and projection (UMAP) (McInnes et al., 2018), distort global topology, while those prioritizing global coherence, such as principal component analysis (PCA) and diffusion maps (Moon et al., 2018; Becht et al., 2019), may obscure fine-grained cell states. Graph-based approaches (Hetzel et al., 2021; Nguyen et al., 2022) and gene co-expression modeling (Deng et al., 2025; Li et al., 2025; Song T. et al., 2022) partially address this by explicitly encoding functional relationships, yet systematic benchmarking reveals that most methods still sacrifice one scale for the other (Tian et al., 2019).

These challenges intensify across modalities: single-cell ATAC sequencing (scATAC-seq) exhibits 90%–95% zero rates versus 60%–80% in single-cell RNA sequencing (scRNA-seq) (Chen et al., 2019; Fang et al., 2021), thus requiring flexible architectures that generalize without extensive re-engineering (Song Q. et al., 2022; Zhao et al., 2024). Hyperbolic geometry offers a principled solution as its exponential volume growth naturally accommodates tree-like hierarchies common in developmental biology (Nickel and Kiela, 2017; Chami et al., 2019; Sarkar, 2012; Bronstein et al., 2021). Existing hyperbolic deep learning methods have improved visualization and captured cellular relationships (Tian et al., 2023; Klimovskaia et al., 2020), but they constrain entire latent spaces to hyperbolic manifolds (Mathieu et al., 2019; Nagano et al., 2019), thus sacrificing compatibility with standard neural architectures and downstream analytical tools. This failure stems from representational constraints: embedding N nodes in a balanced binary tree requires O(N) Euclidean dimensions to avoid distortion, whereas hyperbolic space requires only O(log⁡N) dimensions.

We introduce the Lorentz-regularized variational autoencoder (LiVAE), which applies hyperbolic geometry as regularization over standard Euclidean latent representations rather than constraining the latent space itself. LiVAE learns a primary embedding $z\in R^{d}$ optimized for reconstruction, while a bottleneck (BN) pathway compresses z to $l_{e}\in R^{d_{c}}$ (where $d_{c}\ll d$ ) and reconstructs $l_{d}\in R^{d}$ . A geometric loss enforces that Lorentzian distances—computed after projecting to the hyperboloid model (Ganea et al., 2018; Skopek et al., 2019)—between z and $l_{d}$ remain small, preserving hierarchical structure while maintaining compatibility with downstream tools. This design resolves the local–global tension through complementary objectives: the information bottleneck (Tishby and Zaslavsky, 2015; Strouse and Schwab, 2017) discards technical noise while retaining biological structure, implicitly promoting cross-sample integration (Lopez et al., 2018; Lynch et al., 2023), while the dual reconstruction paths balance local fidelity (primary path from z ) with global coherence (bottleneck path via $l_{d}$ ).

We validate LiVAE on 135 datasets spanning scRNA-seq and scATAC-seq against 21 baseline methods using 12 metrics assessing embedding fidelity, manifold geometry, and robustness. LiVAE consistently achieves superior global topology preservation and noise resilience while remaining competitive on local structure metrics. Component-wise interpretability analysis demonstrates that latent dimensions decompose into biologically meaningful axes corresponding to cell cycle, immune identity, and differentiation programs (Choi et al., 2023; Madrigal et al., 2024).

Our contributions are threefold: (1) architectural innovation: a hybrid design applying hyperbolic regularization to Euclidean representations via information bottlenecks, balancing local fidelity with global coherence; (2) cross-modality generalization: a unified framework handling scRNA-seq and scATAC-seq through modality-appropriate likelihoods without architectural changes; and (3) biological interpretability: latent dimensions aligned with known biological processes that enable mechanistic hypothesis generation beyond black-box embeddings. By resolving the local–global trade-off through geometric regularization, LiVAE provides a flexible foundation for single-cell multi-omics analysis that preserves biological hierarchy without sacrificing compatibility with existing computational workflows.

## 2 Materials and methods

### 2.1 Notation

Throughout this section, $X\in R^{N\times D}$ denotes a batch of N cells with D features, $x_{i}\in R^{D}$ denotes a single cell, and $x_{ij}$ denotes a scalar value (gene or peak j in cell i ). Latent representations include $Z\in R^{N\times d}$ (batch) and $z_{i}\in R^{d}$ (single cell). The dimensions are d (latent dimension) and $d_{c}$ (bottleneck dimension, where $d_{c}\ll d$ ). Lorentzian projections are denoted as $z_{H},l_{dH}\in H^{d+1}$ (hyperboloid manifold).

### 2.2 LiVAE architecture overview

**FIGURE 1:**
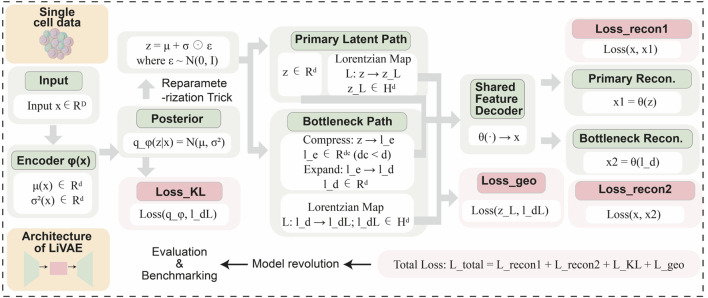
LiVAE architecture. Input x is encoded to latent z , which is processed via two pathways: (1) primary path: direct Lorentzian projection $\left(z_{H}\right)$ ; (2) bottleneck path: compression to $l_{e}$ (dimension $d_{c}\ll d$ ), expansion to $l_{d}$ , and Lorentzian projection to $\left(l_{dH}\right)$ . Shared decoder θ reconstructs from both z and $l_{d}$ . The total loss combines two reconstruction terms $\left(L_{\text{recon}1},L_{\text{recon}2}\right)$ , KL divergence $\left(L_{\text{KL}}\right)$ , and geometric loss $\left(L_{\text{geo}}\right)$ enforcing Lorentzian distance preservation.

LiVAE is a variational autoencoder that applies Lorentzian geometric regularization across a dual-pathway latent space architecture (Figure 1). The encoder ϕ maps the input $x\in R^{D}$ to a diagonal Gaussian posterior $q_{\phi }\left(z|x\right)=N\left(\mu ,\text{diag}\left(\sigma^{2}\right)\right)$ . A latent vector $z\in R^{d}$ is sampled via reparameterization and processed through two parallel pathways: (1) the *primary path* uses z directly for reconstruction and geometric comparison; (2) the *bottleneck path* compresses z to $l_{e}\in R^{d_{c}}$ (where $d_{c}\ll d$ ) and expands it back to $l_{d}\in R^{d}$ . A shared decoder θ reconstructs from both the representations, yielding $x_{1}=\theta \left(z\right)$ and $x_{2}=\theta \left(l_{d}\right)$ .

The model is trained via total loss $L_{\text{total}}$ comprising two reconstruction losses $\left(L_{\text{recon}1},L_{\text{recon}2}\right)$ , Kullback–Leibler (KL) divergence $\left(L_{\text{KL}}\right)$ , and a geometric loss $\left(L_{\text{geo}}\right)$ that enforces Lorentzian distance preservation between z and $l_{d}$ after mapping to the hyperbolic space.

### 2.3 Model architecture

#### 2.3.1 Encoder network

For each cell i in batch $X\in R^{N\times D}$ , the encoder outputs the mean $\mu_{i}\in R^{d}$ and log-variance $\log\sigma_{i}^{2}\in R^{d}$ (Equation 1). Latent vectors are sampled as follows:

$$z_{i}=\mu_{i}+\sigma_{i}⊙\epsilon_{i},\;\text{where }\epsilon_{i}∼N\left(0,I\right),$$ (1)

and $\sigma_{i}=\exp\left(\frac{1}{2}\log\sigma_{i}^{2}\right)$ . The encoder consists of a single hidden layer with 128 units, ReLU activation, and layer normalization.

#### 2.3.2 Dual latent pathways and decoder

The bottleneck path applies two linear transformations: compression $L_{e}=ZW_{e}+b_{e}$ (to dimension $d_{c}$ ) and expansion $L_{d}=L_{e}W_{d}+b_{d}$ (back to dimension d ), where $W_{e}\in R^{d\times d_{c}}$ and $W_{d}\in R^{d_{c}\times d}$ , producing compressed representation $L_{e}\in R^{N\times d_{c}}$ and expanded representation $L_{d}\in R^{N\times d}$ .

The decoder mirrors the encoder architecture, outputting distribution parameters via linear layers followed by softmax normalization. It generates two reconstructions:

- Primary reconstruction: $X_{1}=\theta \left(Z\right)$ .
- Bottleneck reconstruction: $X_{2}=\theta \left(L_{d}\right)$ .

### 2.4 Loss function

The total loss function (Equation 2) is a weighted sum of four components:

$$L_{\text{total}}=L_{\text{recon}1}+L_{\text{recon}2}+\lambda_{\text{geo}}L_{\text{geo}}+\beta L_{\text{KL}},$$ (2)

where $\lambda_{\text{geo}}\geq 0$ and β≥0 are hyperparameters balancing the regularization terms.

#### 2.4.1 Reconstruction losses

The reconstruction losses (Equation 3) measure how well each pathway captures the input:

$$L_{\text{recon}1}=-E_{q_{\phi }}\left[\log p\left(X|Z\right)\right],\;L_{\text{recon}2}=-E_{q_{\phi }}\left[\log p\left(X|L_{d}\right)\right].$$ (3)

The likelihood p(X|⋅) is modality-specific.

- scRNA-seq: negative binomial (NB) distribution with the mean $\mu_{ij}$ (decoder output scaled by the cell library size) and gene-specific dispersion parameter $\theta_{j}$ to model count overdispersion.
- scATAC-seq: zero-inflated negative binomial (ZINB) with additional zero-inflation probability $\pi_{ij}$ from a separate decoder head, accounting for excess zeros in chromatin accessibility (90%–95% sparsity vs. 60%–80% in scRNA-seq).
- Alternative: Poisson and zero-inflated Poisson (ZIP) likelihoods are also supported for datasets with minimal overdispersion.

For all likelihoods, the predicted means are obtained as $\mu_{ij}=\text{softmax}{\left(\text{decoder}\left(\cdot \right)\right)}_{ij}\cdot \sum_{k}x_{ik}$ , where the decoder output is normalized by the cell-wise library size.

#### 2.4.2 Kullback–Leibler divergence

The KL divergence is the standard variational autoencoder (VAE) regularizer that encourages the posterior to approximate a unit Gaussian prior (Equation 4):

$$L_{\text{KL}}=\frac{1}{2Nd}\overset{N}{\underset{i=1}{\sum }}\overset{d}{\underset{j=1}{\sum }}\left(\sigma_{ij}^{2}+\mu_{ij}^{2}-1-\log\sigma_{ij}^{2}\right).$$ (4)

#### 2.4.3 Geometric loss

The geometric loss enforces that the bottleneck transformation preserves the hyperbolic geometric structure. Euclidean vectors z and $l_{d}$ are mapped to the hyperboloid $H^{d+1}$ (the Lorentzian model of hyperbolic space) via the exponential map at origin o=(1,0,…,0) (Equation 5):

$$\exp_{o}\left(v\right)=\left\{\begin{array}{cc} \left(\cosh\left(\left\|v\right\|\right),\sinh\left(\left\|v\right\|\right)\cdot \frac{v}{\left\|v\right\|}\right),\; & \|v\|>0,\\ \left(1,0\right),\; & \|v\|=0, \end{array}\right.$$ (5)

where $v=\left(0,v_{1},\ldots ,v_{d}\right)\in T_{o}H^{d+1}$ is a tangent vector with the first coordinate zero.

The geometric loss is the mean squared Lorentzian distance between paired representations (Equation 6):

$$L_{\text{geo}}=\frac{1}{N}\overset{N}{\underset{i=1}{\sum }}d_{H}{\left(z_{H,i},l_{dH,i}\right)}^{2},$$ (6)

where $z_{H}=\exp_{o}\left(z\right)$ and $l_{dH}=\exp_{o}\left(l_{d}\right)$ , and the Lorentzian distance is obtained as follows (Equation 7):

$$d_{H}\left(u,v\right)=\text{arccosh}\left(-{\left\langle u,v\right\rangle }_{L}\right),$$ (7)

with the Lorentzian inner product ${\left\langle u,v\right\rangle }_{L}=-u_{0}v_{0}+\sum_{k=1}^{d}u_{k}v_{k}$ . For numerical stability, we use $d_{H}=\log\left(2\alpha \right)$ when $\alpha =-{\left\langle u,v\right\rangle }_{L}>10^{4}$ , with clamping $\alpha \geq 1+10^{-8}$ .

### 2.5 Evaluation metrics

We assess LiVAE performance using 12 metrics organized into four categories, evaluating complementary aspects of representation quality.

#### 2.5.1 Clustering quality metrics

We assess the biological population structure using five standard metrics and one novel metric:

Standard metrics: Normalized mutual information (NMI) and adjusted Rand index (ARI) measure agreement between the predicted clusters and ground-truth cell-type labels, with values near 1 indicating strong correspondence. Average silhouette width (ASW) and the Calinski–Harabasz index (CAL) quantify cluster cohesion and separation (higher is better), while the Davies–Bouldin index (DAV) measures the average cluster similarity (lower is better). These metrics are computed using standard implementations in scikit-learn.

Coupling degree (COR) (Equation 8): We introduce this metric to quantify preservation of interdependent biological programs:

$$\text{COR}=\frac{1}{k\left(k-1\right)}\overset{k}{\underset{i=1}{\sum }}\overset{k}{\underset{j\neq i}{\sum }}|\rho_{ij}|,$$ (8)

where $\rho_{ij}$ is the Pearson correlation between latent dimensions i and j and k is the latent space dimensionality. Higher COR values indicate stronger coupling, reflecting coordinated gene expression programs that are essential for continuous differentiation trajectories.

#### 2.5.2 Dimensionality reduction embedding quality metrics

We evaluate how effectively latent representations Z project to interpretable low-dimensional spaces (UMAP and t-SNE) while preserving biological relationships.

Distance correlation $\left(\rho_{\text{dist}}\right)$ (Equation 9) quantifies the preservation of pairwise distance relationships:

$$\rho_{\text{dist}}=\rho_{\text{Spearman}}\left(\text{vec}\left(D_{Z}\right),\text{vec}\left(D_{E}\right)\right),$$ (9)

where $D_{Z}$ and $D_{E}$ are pairwise Euclidean distance matrices in latent and embedding spaces, respectively, vec(⋅) vectorizes the upper triangle, and $\rho_{\text{Spearman}}$ is the Spearman rank correlation coefficient. Higher values indicate better preservation of the global structure.

Local quality $\left(Q_{\text{local}}\right)$ and global quality $\left(Q_{\text{global}}\right)$ (Equation 10) measure preservation at different scales through co-ranking matrix analysis:

$$Q_{\text{local}}=\frac{1}{K_{\max}}\overset{K_{\max}}{\underset{K=1}{\sum }}Q_{NX}\left(K\right),\;Q_{\text{global}}=\frac{1}{n-1-K_{\max}}\overset{n-1}{\underset{K=K_{\max}+1}{\sum }}Q_{NX}\left(K\right),$$ (10)

where $Q_{NX}\left(K\right)$ is the normalized co-ranking quality measure at neighborhood size K and $K_{\max}$ is the optimal local neighborhood boundary. Higher values indicate better maintenance of local neighborhoods and large-scale topology, respectively.

Overall embedding quality $\left(Q_{\text{embed}}\right)$ (Equation 11) combines the three components:

$$Q_{\text{embed}}=\frac{1}{3}\left(\rho_{\text{dist}}+Q_{\text{local}}+Q_{\text{global}}\right).$$ (11)

#### 2.5.3 Intrinsic manifold quality metrics

We characterize geometric properties of the latent manifold through spectral analysis of the covariance matrix $C=\frac{1}{n-1}Z^{T}Z$ , with eigenvalues $\lambda_{1}\geq \ldots \geq \lambda_{k}$ .

Manifold dimensionality $\left(M_{\text{dim}}\right)$ (Equation 12) measures representation compactness:

$$M_{\text{dim}}=1-\frac{d_{\text{eff}}-1}{k-1},$$ (12)

where $d_{\text{eff}}$ represents the number of principal components explaining 95% variance. Higher values indicate more efficient encoding.

Spectral decay rate $\left(S_{\text{decay}}\right)$ (Equation 13) quantifies hierarchical structure clarity:

$$S_{\text{decay}}=\frac{1}{1+e^{\left|\beta \right|}}\cdot \frac{\lambda_{1}}{\sum_{i=1}^{k}\lambda_{i}},$$ (13)

where β is the slope from log-linear regression on eigenvalues. Higher values indicate steeper spectrum decay, reflecting clear hierarchical organization.

Participation ratio $\left(P_{\text{ratio}}\right)$ (Equation 14) assesses the balance of variance distribution:

$$P_{\text{ratio}}=\frac{1}{k}\cdot \frac{{\left(\sum_{i=1}^{k}\lambda_{i}\right)}^{2}}{\sum_{i=1}^{k}\lambda_{i}^{2}}.$$ (14)

Higher values indicate more uniform utilization of latent dimensions, thereby preventing dimension collapse.

Anisotropy score $\left(A_{\text{score}}\right)$ (Equation 15) quantifies directional bias strength:

$$A_{\text{score}}=\tanh\left(\frac{\log\left(\lambda_{1}\right)-\log\left(\lambda_{k}+\epsilon \right)}{4}\right),$$ (15)

where $\epsilon =10^{-8}$ . Higher values indicate stronger directional structure along the dominant axes, which is essential for trajectory representation.

Trajectory directionality $\left(T_{\text{dir}}\right)$ (Equation 16) measures dominance of the primary variation axis:

$$T_{\text{dir}}=\frac{\lambda_{1}}{\sum_{i=2}^{k}\lambda_{i}+\epsilon }.$$ (16)

Higher values indicate a single dominant trajectory, which is characteristic of linear differentiation processes.

Noise resilience $\left(N_{\text{res}}\right)$ (Equation 17) approximates the signal-to-noise ratio:

$$N_{\text{res}}=\min\left(\frac{\sum_{i=1}^{2}\lambda_{i}}{\sum_{i=3}^{k}\lambda_{i}+\epsilon }\cdot \frac{1}{10},1\right).$$ (17)

Higher values indicate robust separation between signal and noise subspaces.

Composite scores.

We define two summary metrics: core intrinsic quality (Equation 18) integrates the fundamental geometric properties,

$$Q_{\text{core}}=\frac{1}{4}\left(M_{\text{dim}}+S_{\text{decay}}+P_{\text{ratio}}+A_{\text{score}}\right),$$ (18)

while overall intrinsic quality (Equation 19) incorporates task-oriented components with weights (α,β,γ)=(0.5,0.3,0.2) :

$$Q_{\text{overall}}=\alpha \cdot Q_{\text{core}}+\beta \cdot T_{\text{dir}}+\gamma \cdot N_{\text{res}}.$$ (19)

#### 2.5.4 Batch integration quality

The integration local inverse Simpson index (iLISI) (Equation 20) measures batch mixing quality. For each cell i ,

$${\text{iLISI}}_{i}=\frac{1}{\sum_{j=1}^{B}p_{ij}^{2}},$$ (20)

where B represents the number of batches and $p_{ij}$ represents the proportion of cell i ’s k -nearest neighbors from batch j , and it is computed using a Gaussian kernel with the bandwidth determined by perplexity. The overall score is $\text{iLISI}=\frac{1}{n}\sum_{i=1}^{n}{\text{iLISI}}_{i}$ . Higher values (approaching B ) indicate better batch integration, while values near 1 indicate poor mixing.

### 2.6 Datasets and preprocessing

#### 2.6.1 Dataset selection

We curated 135 single-cell datasets from public repositories (Gene Expression Omnibus, GEO): 53 scRNA-seq dataset and 82 scATAC-seq dataset samples. Raw single-cell count matrices underwent quality control and normalization prior to model training. Both modalities require raw integer counts as input as the model employs count-based likelihood functions.

#### 2.6.2 scRNA-seq preprocessing

The top 5,000 highly variable genes (HVGs) were selected by modeling the mean–variance relationship in count data. For model input, normalized data were obtained by applying log(x+1) transformation, followed by z -score standardization with outlier clipping at ±10 standard deviations.

#### 2.6.3 scATAC-seq preprocessing

Term frequency–inverse document frequency (TF-IDF) normalization (Equation 21) was applied:

$${\text{TF}-\text{IDF}}_{ij}={\text{TF}}_{ij}\times {\text{IDF}}_{j}\times s,$$ (21)

where the term frequency for cell i and peak j is ${\text{TF}}_{ij}=x_{ij}/\sum_{k}x_{ik}$ , the inverse document frequency is ${\text{IDF}}_{j}=\log\left(1+\frac{N}{n_{j}}\right)$ , with N being the total number of cells and $n_{j}$ being the number of cells where peak j is accessible, and $s=10^{4}$ is a scaling factor. Highly variable peaks (HVPs) were identified using variance-based selection on TF-IDF normalized values, which were restricted to peaks with accessibility between 1% and 95% of cells. The top 10,000 HVPs were selected as input features.

### 2.7 Model hyperparameters

LiVAE was configured with the following default hyperparameters. The encoder and decoder networks each contained a single hidden layer of dimension 128. The latent space dimension was set to d=10 , and the information bottleneck dimension was set to $d_{c}=2$ . The loss function weights were the primary reconstruction weight $\lambda_{\text{recon}1}=1.0$ , bottleneck reconstruction weight $\lambda_{\text{recon}2}=1.0$ , KL divergence weight β=1.0 , and geometric loss weight $\lambda_{\text{geo}}=5.0$ . For reconstruction losses, we employed NB likelihood for scRNA-seq data and ZINB likelihood for scATAC-seq data. Training was performed using the Adam optimizer with a learning rate of $1\times 10^{-4}$ and a batch size of 128. Gradient clipping with a threshold of 1.0 was applied. Layer normalization was employed in both the encoder and decoder networks.

### 2.8 Baseline methods

We compared LiVAE against 21 methods spanning four categories:

- Classical dimensionality reduction (seven methods): PCA, kernel PCA (KPCA), factor analysis (FA), non-negative matrix factorization (NMF), independent component analysis (ICA), truncated SVD (TSVD), and dictionary learning (DICL).
- Deep generative models (eight methods): standard VAE, β -variational autoencoder ( β -VAE), total correlation β -VAE ( β -TCVAE), disentangled inferred prior VAE (DIPVAE), information maximizing VAE (InfoVAE), single-cell variational inference (scVI), single-cell deep clustering (scDeepCluster), single-cell deep hyperbolic manifold learning (scDHMap), and single-cell trajectory optimization (scTour).
- Graph and contrastive learning (three methods): contrastive learning for scRNA-seq (CLEAR), single-cell graph neural network (scGNN), and single-cell graph contrastive clustering (scGCC).
- Modality-specific methods (three methods): latent semantic indexing (LSI), peak variational inference (PeakVI), and Poisson variational inference (PoissonVI) for scATAC-seq.

### 2.9 Statistical analysis

We used paired experimental designs (identical datasets for all methods). For each metric, normality was assessed using the Shapiro–Wilk test (α=0.05) . Multi-method comparisons employed repeated measures analysis of variance (ANOVA) (normal data) or Friedman test (non-normal), followed by Tukey honest significant difference (HSD) or Bonferroni-corrected Wilcoxon signed-rank *post hoc* tests, respectively. The significance levels were * p<0.05 , ** p<0.01 , and *** p<0.001 .

## 3 Results

### 3.1 Architectural progression from foundational VAEs yields comprehensive performance gains

**FIGURE 2:**
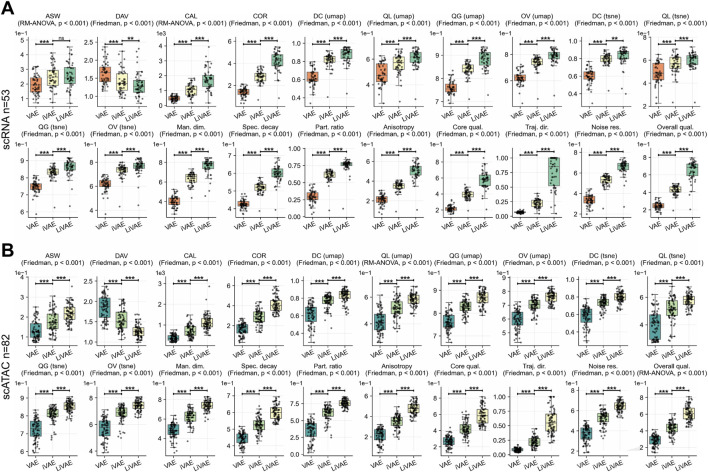
Progressive architectural enhancements yield consistent performance gains. Boxplots display performance differences ( Δ ; LiVAE − baseline) across five evaluation categories. Statistical significance was assessed using Tukey’s HSD *post hoc* test for ASW and CAL (scRNA-seq), Q local (UMAP), and overall intrinsic quality (scATAC-seq); Bonferroni-corrected Wilcoxon signed-rank tests were applied for all other metrics. Boxes indicate the median and IQR; whiskers extend to 1.5× IQR. Statistical methods are indicated in subplot titles. **(A)** scRNA-seq datasets (n=53) . **(B)** scATAC-seq datasets (n=82) .

We benchmarked LiVAE against its foundational predecessors—standard VAE and information bottleneck VAE (iVAE)—using 135 datasets (53 scRNA-seq and 82 scATAC-seq). LiVAE’s complete architecture established a new performance baseline, delivering statistically significant improvements across nearly all metrics for both scRNA-seq (Figure 2A) and scATAC-seq datasets (Figure 2B; Table 1).

**TABLE 1:** Performance differences between LiVAE and baseline VAE models across scRNA-seq (n=53) and scATAC-seq (n=82) datasets. Values represent absolute differences ( Δ ; LiVAE − baseline). Significance levels: ^***^ p<0.001 , ^**^ p<0.01 , and ^*^ p<0.05 .

Metric	scRNA-seq		scATAC-seq	
	vs. VAE	vs. iVAE	vs. VAE	vs. iVAE
*Clustering quality*				
*ASW*	$0.063^{***}$	0.009	$0.086^{***}$	$0.041^{***}$
*DAV*	$-0.311^{***}$	$-0.079^{**}$	$-0.575^{***}$	$-0.276^{***}$
*CAL*	$1270.811^{***}$	$718.553^{***}$	$754.828^{***}$	$424.693^{***}$
*COR*	$2.802^{***}$	$1.395^{***}$	$2.479^{***}$	$1.232^{***}$
*UMAP embedding fidelity*				
*Dist. corr*	$0.251^{***}$	$0.062^{***}$	$0.217^{***}$	$0.065^{***}$
*Q local*	$0.127^{***}$	$0.047^{***}$	$0.165^{***}$	$0.065^{***}$
*Q global*	$0.123^{***}$	$0.044^{***}$	$0.105^{***}$	$0.039^{***}$
*Overall qual*	$0.167^{***}$	$0.051^{***}$	$0.162^{***}$	$0.056^{***}$
*t-SNE embedding fidelity*				
*Dist. corr*	$0.229^{***}$	$0.036^{**}$	$0.221^{***}$	$0.067^{***}$
*Q local*	$0.111^{***}$	$0.033^{***}$	$0.178^{***}$	$0.066^{***}$
*Q global*	$0.113^{***}$	$0.031^{***}$	$0.130^{***}$	$0.045^{***}$
*Overall qual*	$0.151^{***}$	$0.033^{***}$	$0.176^{***}$	$0.059^{***}$
*Latent manifold structure*				
*Manif. dim*	$0.356^{***}$	$0.123^{***}$	$0.258^{***}$	$0.117^{***}$
*Spectral decay*	$0.170^{***}$	$0.078^{***}$	$0.159^{***}$	$0.076^{***}$
*Part. ratio*	$0.467^{***}$	$0.149^{***}$	$0.416^{***}$	$0.149^{***}$
*Anisotropy*	$0.287^{***}$	$0.153^{***}$	$0.266^{***}$	$0.129^{***}$
*Intrinsic quality and robustness*				
*Core intrin. qual*	$0.320^{***}$	$0.126^{***}$	$0.275^{***}$	$0.117^{***}$
*Traj. directionality*	$0.356^{***}$	$0.181^{***}$	$0.318^{***}$	$0.160^{***}$
*Noise resilience*	$0.678^{***}$	$0.526^{***}$	$0.486^{***}$	$0.350^{***}$
*Overall intrin. qual*	$0.403^{***}$	$0.222^{***}$	$0.330^{***}$	$0.177^{***}$

The most striking advantage was a profound increase in model robustness. For scRNA-seq (Figure 2A), LiVAE boosted noise resilience by Δ=0.678 (vs. VAE) and Δ=0.526 (vs. iVAE), translating to superior overall intrinsic quality ( Δ=0.403 and Δ=0.222 , respectively; all p<0.001 ). Comparable patterns were obtained for scATAC-seq (Figure 2B), with noise resilience gains of Δ=0.486 and Δ=0.350 , and overall intrinsic quality improvements of Δ=0.330 and Δ=0.177 .

This enhanced robustness stemmed from LiVAE’s geometrically expressive latent space. For scRNA-seq, the participation ratio increased by Δ=0.467 (vs. VAE) and Δ=0.149 (vs. iVAE), while manifold dimensionality, spectral decay, and anisotropy improved by Δ=0.356/0.123 , Δ=0.170/0.078 , and Δ=0.287/0.153 , respectively. Similar gains were obtained for scATAC-seq (Table 1). These structural improvements enabled faithful visualizations, with UMAP distance correlation increasing by Δ=0.251/0.062 (scRNA-seq) and Δ=0.217/0.065 (scATAC-seq). Clustering performance improved dramatically; for scRNA-seq, cluster alignment (CAL) increased by Δ=1270.811 (vs. VAE) and Δ=718.553 (vs. iVAE), while latent dimension coupling (COR) increased by Δ=2.802 and Δ=1.395 (all p<0.001 ), indicating stronger preservation of coordinated biological programs. Collectively, these results demonstrate that LiVAE’s components—bottleneck, dual-pathway loss, and Lorentz regularization—synergistically create a more stable, powerful, and biologically informative model.

### 3.2 Balanced profile of local fidelity and global structure against classical methods

We benchmarked LiVAE against seven classical algorithms on n=53 scRNA-seq datasets. While some classical methods showed competitive local neighborhood preservation, LiVAE provided superior global coherence, manifold complexity, and robustness (Table 2).

**TABLE 2:** Performance differences between LiVAE and classical dimensionality reduction methods across n=53 scRNA-seq datasets. Values represent absolute differences ( Δ ; LiVAE − baseline). Significance levels: ^***^ p<0.001 , ^**^ p<0.01 , and ^*^ p<0.05 .

Metric	PCA	KPCA	ICA	FA	NMF	TSVD	DICL
*UMAP embedding fidelity*							
*Dist. corr*	$0.215^{***}$	$0.216^{***}$	$0.436^{***}$	$0.345^{***}$	$0.291^{***}$	$0.209^{***}$	$0.280^{***}$
*Q local*	0.025	0.025	$0.072^{***}$	0.029	0.002	0.027	$-0.036^{***}$
*Q global*	$0.100^{***}$	$0.099^{***}$	$0.177^{***}$	$0.137^{***}$	$0.122^{***}$	$0.098^{***}$	$0.122^{***}$
*Overall qual*	$0.113^{***}$	$0.113^{***}$	$0.228^{***}$	$0.170^{***}$	$0.138^{***}$	$0.111^{***}$	$0.122^{***}$
*t-SNE embedding fidelity*							
*Dist. corr*	$0.173^{***}$	$0.174^{***}$	$0.383^{***}$	$0.320^{***}$	$0.237^{***}$	$0.172^{***}$	$0.248^{***}$
*Q local*	−0.001	0.000	$0.034^{**}$	−0.005	$-0.034^{*}$	0.001	$-0.079^{***}$
*Q global*	$0.079^{***}$	$0.078^{***}$	$0.155^{***}$	$0.119^{***}$	$0.098^{***}$	$0.079^{***}$	$0.103^{***}$
*Overall qual*	$0.084^{***}$	$0.084^{***}$	$0.191^{***}$	$0.145^{***}$	$0.100^{***}$	$0.084^{***}$	$0.091^{***}$
*Latent manifold structure*							
*Manif. dim*	$0.241^{***}$	$0.241^{***}$	$0.426^{***}$	$0.467^{***}$	$0.371^{***}$	$0.251^{***}$	$0.384^{***}$
*Spectral decay*	$0.128^{***}$	$0.128^{***}$	$0.258^{***}$	$0.255^{***}$	$0.154^{***}$	$0.124^{***}$	$0.155^{***}$
*Part. ratio*	$0.319^{***}$	$0.319^{***}$	$0.761^{***}$	$0.760^{***}$	$0.444^{***}$	$0.310^{***}$	$0.451^{***}$
*Anisotropy*	$0.259^{***}$	$0.259^{***}$	$0.494^{***}$	$0.489^{***}$	$0.190^{***}$	$0.240^{***}$	$0.181^{***}$
*Intrinsic quality and robustness*							
*Core intrin. qual*	$0.237^{***}$	$0.237^{***}$	$0.485^{***}$	$0.493^{***}$	$0.290^{***}$	$0.231^{***}$	$0.293^{***}$
*Traj. directionality*	$0.252^{***}$	$0.252^{***}$	$0.479^{***}$	$0.476^{***}$	$0.339^{***}$	$0.248^{***}$	$0.333^{***}$
*Noise resilience*	$0.622^{***}$	$0.622^{***}$	$0.712^{***}$	$0.711^{***}$	$0.661^{***}$	$0.620^{***}$	$0.663^{***}$
*Overall intrin. qual*	$0.318^{***}$	$0.318^{***}$	$0.528^{***}$	$0.531^{***}$	$0.379^{***}$	$0.314^{***}$	$0.379^{***}$

**FIGURE 3:**
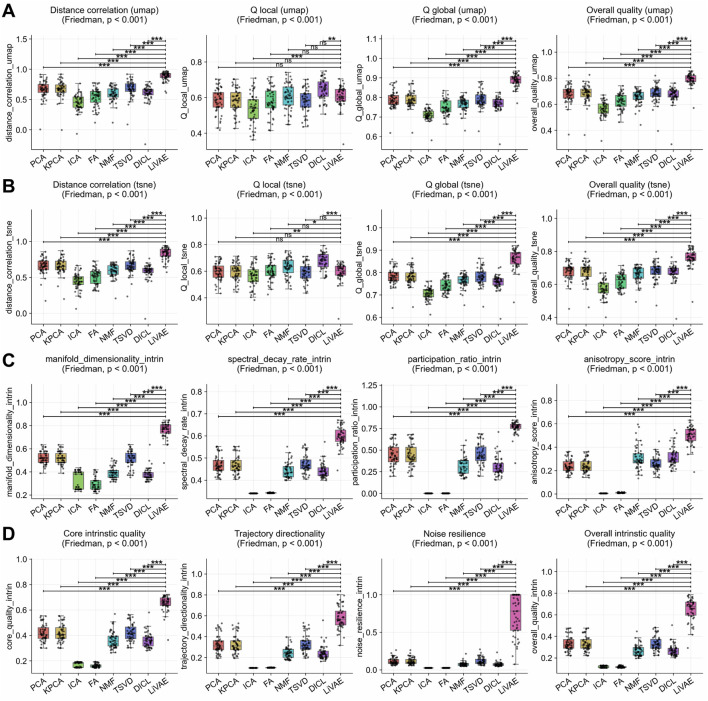
Balanced local–global performance relative to classical dimensionality reduction. Boxplots summarize metric distributions for LiVAE and seven classical baselines across n=53 scRNA-seq datasets. Boxes indicate the median and IQR; whiskers extend to 1.5× IQR. Statistical significance was assessed using Bonferroni-corrected Wilcoxon tests (α=0.0018) . **(A)** UMAP embedding fidelity. **(B)** t-SNE embedding fidelity. **(C)** Latent manifold structure. **(D)** Intrinsic quality and robustness.

LiVAE’s UMAP local quality (Figure 3A) was statistically equivalent to those of PCA, KPCA, FA, NMF, and TSVD but slightly lower than that of dictionary learning (DICL; Δ=−0.036 , p<0.001 ) and ICA (Δ=0.072) . However, LiVAE demonstrated massive advantages in global structure, with UMAP distance correlation surpassing all methods by Δ=0.209 (vs. TSVD) to Δ=0.436 (vs. ICA), yielding overall UMAP quality gains of Δ=0.111 to Δ=0.228 (all p<0.001 ). Parallel trends emerged for t-SNE (Figure 3B), with distance correlation improvements of Δ=0.172 to Δ=0.383 and overall quality gains of Δ=0.084 to Δ=0.191 .

This superior organization reflected a sophisticated latent space (Figure 3C). All four manifold metrics improved substantially: dimensionality ( Δ=0.241 to Δ=0.467 ), spectral decay ( Δ=0.124 to Δ=0.258 ), participation ratio ( Δ=0.310 to Δ=0.761 ), and anisotropy ( Δ=0.181 to Δ=0.494 ). Critically, noise resilience exceeded that of all classical methods ( Δ=0.620 to Δ=0.712 ; Figure 3D), culminating in overall intrinsic quality gains of Δ=0.314 to Δ=0.531 (all p<0.001 ). Thus, LiVAE delivers a balanced, robust solution for single-cell exploratory analysis.

### 3.3 Competitive edge in stability and manifold quality against state-of-the-art generative models

We assessed LiVAE against eight state-of-the-art deep generative models on n=53 scRNA-seq datasets, confirming it as a top-tier general-purpose embedding method distinguished by exceptional stability and global latent-space integrity (Table 3).

**TABLE 3:** Performance differences between LiVAE and advanced generative and scRNA-seq-specialized methods across n=53 scRNA-seq datasets. Values represent absolute differences ( Δ ; LiVAE − baseline). Significance levels: ^***^ p<0.001 , ^**^ p<0.01 , and ^*^ p<0.05 .

Metric	β -VAE	β -TCVAE	DIPVAE	InfoVAE	scVI	scDeepCluster	scDHMap	scTour
*UMAP embedding fidelity*								
*Dist. corr*	$0.411^{***}$	$0.294^{***}$	$0.329^{***}$	$0.256^{***}$	$0.453^{***}$	$0.289^{***}$	$0.176^{***}$	−0.020
*Q local*	$0.271^{***}$	$0.188^{***}$	$0.136^{***}$	$0.133^{***}$	$0.176^{***}$	$0.165^{***}$	−0.042	$0.024^{*}$
*Q global*	$0.170^{***}$	$0.141^{***}$	$0.160^{***}$	$0.126^{***}$	$0.186^{***}$	$0.130^{***}$	$0.083^{***}$	−0.008
*Overall qual*	$0.284^{***}$	$0.208^{***}$	$0.208^{***}$	$0.172^{***}$	$0.272^{***}$	$0.195^{***}$	$0.072^{***}$	−0.001
*t-SNE embedding fidelity*								
*Dist. corr*	$0.393^{***}$	$0.287^{***}$	$0.346^{***}$	$0.243^{***}$	$0.427^{***}$	$0.268^{***}$	$0.136^{***}$	0.024
*Q local*	$0.271^{***}$	$0.178^{***}$	$0.108^{***}$	$0.112^{***}$	$0.150^{***}$	$0.151^{***}$	$-0.070^{***}$	0.022
*Q global*	$0.190^{***}$	$0.143^{***}$	$0.160^{***}$	$0.120^{***}$	$0.180^{***}$	$0.130^{***}$	$0.071^{***}$	0.008
*Overall qual*	$0.285^{***}$	$0.203^{***}$	$0.205^{***}$	$0.158^{***}$	$0.252^{***}$	$0.183^{***}$	$0.046^{**}$	$0.018^{*}$
*Latent manifold structure*								
*Manif. dim*	$0.386^{***}$	$0.372^{***}$	$0.531^{***}$	$0.360^{***}$	$0.355^{***}$	$0.277^{***}$	0.016	$0.059^{***}$
*Spectral decay*	$0.210^{***}$	$0.193^{***}$	$0.216^{***}$	$0.168^{***}$	$0.221^{***}$	$0.094^{***}$	$-0.042^{***}$	0.009
*Part. ratio*	$0.652^{***}$	$0.570^{***}$	$0.657^{***}$	$0.471^{***}$	$0.698^{***}$	$0.290^{***}$	$0.083^{***}$	0.013
*Anisotropy*	$0.393^{***}$	$0.353^{***}$	$0.129^{***}$	$0.287^{***}$	$0.427^{***}$	$0.085^{***}$	$-0.213^{***}$	$0.036^{*}$
*Intrinsic quality and robustness*								
*Core intrin. qual*	$0.410^{***}$	$0.372^{***}$	$0.383^{***}$	$0.322^{***}$	$0.425^{***}$	$0.186^{***}$	−0.039	$0.030^{**}$
*Traj. directionality*	$0.400^{***}$	$0.377^{***}$	$0.445^{***}$	$0.350^{***}$	$0.415^{***}$	$0.267^{***}$	$0.163^{***}$	−0.007
*Noise resilience*	$0.697^{***}$	$0.686^{***}$	$0.709^{***}$	$0.674^{***}$	$0.702^{***}$	$0.630^{***}$	$0.422^{***}$	$0.184^{***}$
*Overall intrin. qual*	$0.464^{***}$	$0.436^{***}$	$0.467^{***}$	$0.401^{***}$	$0.477^{***}$	$0.299^{***}$	$0.114^{***}$	$0.049^{**}$

**FIGURE 4:**
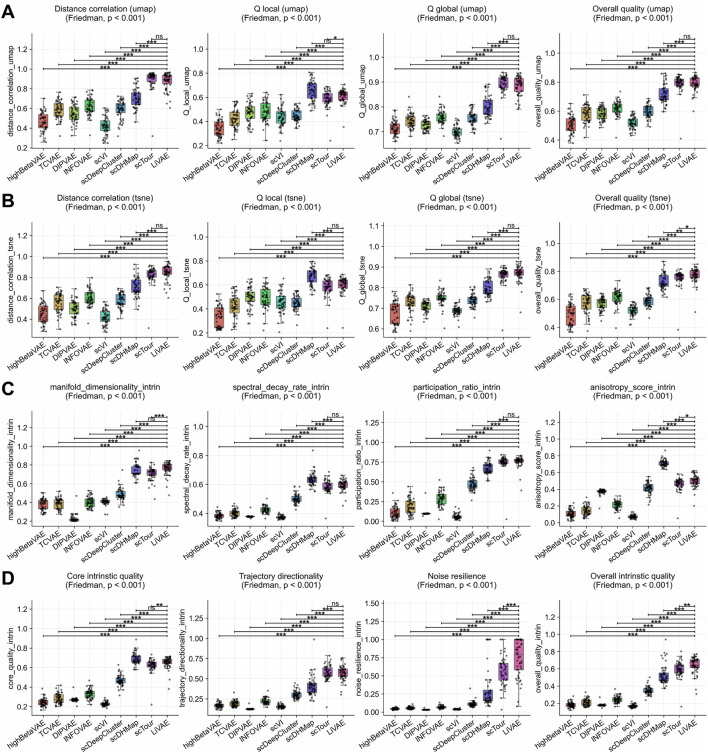
Enhanced global fidelity and stability versus advanced deep generative models. Boxplots compare LiVAE with eight state-of-the-art baselines across n=53 scRNA-seq datasets. Boxes indicate the median and IQR; whiskers extend to 1.5× IQR. Statistical significance was assessed using Bonferroni-corrected Wilcoxon tests (α=0.0014) . **(A)** UMAP embedding fidelity. **(B)** t-SNE embedding fidelity. **(C)** Latent manifold structure. **(D)** Intrinsic quality and robustness.

While trajectory-focused models such as scTour achieved UMAP distance correlation (Figure 4A) statistically equivalent to LiVAE’s ( Δ=−0.020 , n.s.), LiVAE consistently outperformed the majority across nearly all metrics. Its paramount advantage was robustness: noise resilience exceeded all eight competitors by Δ=0.184 (vs. scTour) to Δ=0.709 (vs. DIPVAE; Figure 4D), fostering overall intrinsic quality gains of Δ=0.049 to Δ=0.477 (all p≤0.01 ).

These strengths translated into superior geometric organization. LiVAE delivered UMAP overall quality improvements of Δ=0.072 to Δ=0.284 (Figure 4A), with distance correlation gains of Δ=0.176 to Δ=0.453 (except scTour). The participation ratio consistently exceeded that of all methods ( Δ=0.013 to Δ=0.698 ; Figure 4C), indicating richer manifold complexity. While specialized models such as scDHMap yielded higher anisotropy (Δ=−0.213) , reflecting trajectory optimization, LiVAE provided a state-of-the-art balance of local fidelity (Figures 4A,B), global structure, and best-in-class robustness, making it ideal for broad single-cell data exploration.

### 3.4 Implicit geometric regularization is comparable to explicit graph-based architectures

We benchmarked LiVAE against three prominent graph-aware models (CLEAR, scGNN, and scGCC) on n=53 scRNA-seq datasets. LiVAE’s implicit geometric regularization matched or exceeded explicit graph-based methods, particularly in global fidelity and robustness (Table 4).

**TABLE 4:** Performance differences between LiVAE and graph-based deep learning methods across n=53 scRNA-seq datasets. Values represent absolute differences ( Δ ; LiVAE − baseline). Significance levels: ^***^ p<0.001 , ^**^ p<0.01 , and ^*^ p<0.05 .

Metric	vs. CLEAR	vs. scGNN	vs. scGCC
*UMAP embedding fidelity*			
*Dist. corr*	$0.491^{***}$	$0.470^{***}$	$0.398^{***}$
*Q local*	$0.324^{***}$	$0.082^{***}$	$0.048^{***}$
*Q global*	$0.198^{***}$	$0.167^{***}$	$0.179^{***}$
*Overall qual*	$0.338^{***}$	$0.239^{***}$	$0.208^{***}$
*t-SNE embedding fidelity*			
*Dist. corr*	$0.560^{***}$	$0.418^{***}$	$0.384^{***}$
*Q local*	0.296	$0.037^{***}$	$-0.018^{**}$
*Q global*	$0.269^{***}$	$0.149^{***}$	$0.161^{***}$
*Overall qual*	$0.375^{***}$	$0.201^{***}$	$0.176^{***}$
*Latent manifold structure*			
*Manif. dim*	$0.359^{***}$	$0.018^{***}$	0.281
*Spectral decay*	$0.230^{***}$	$0.054^{***}$	$0.092^{***}$
*Part. ratio*	$0.729^{***}$	$0.019^{***}$	0.348
*Anisotropy*	$0.458^{**}$	$-0.146^{***}$	$0.139^{***}$
*Intrinsic quality and robustness*			
*Core intrin. qual*	$0.444^{***}$	$-0.014^{***}$	0.215
*Traj. directionality*	$0.427^{***}$	$0.280^{***}$	$0.238^{***}$
*Noise resilience*	$0.722^{***}$	$0.600^{***}$	$0.545^{***}$
*Overall intrin. qual*	$0.495^{***}$	$0.197^{***}$	$0.288^{***}$

**FIGURE 5:**
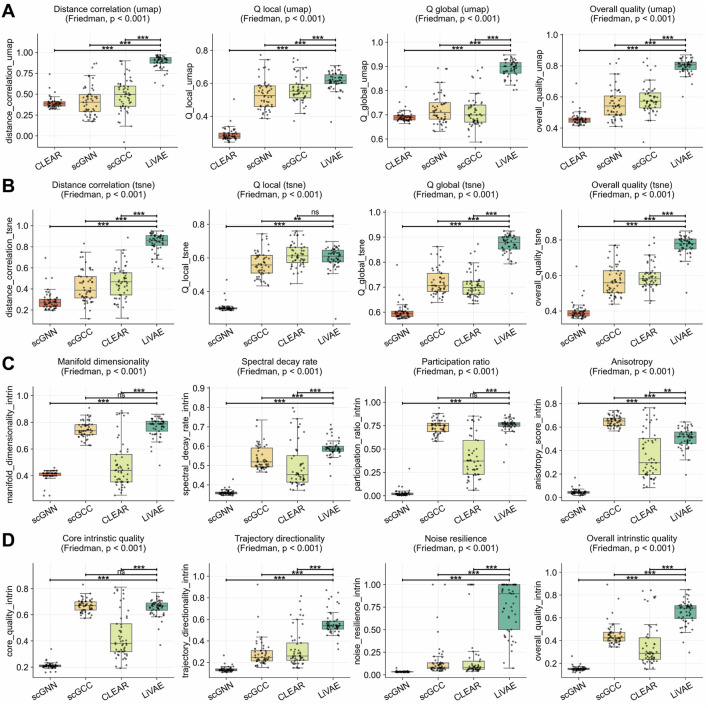
Strong embedding quality and robustness without explicit graph regularization. Boxplots compare LiVAE with three graph-based baselines across n=53 scRNA-seq datasets. Boxes indicate the median and IQR; whiskers extend to 1.5× IQR. Statistical significance was assessed using Bonferroni-corrected Wilcoxon tests (α=0.0083) . **(A)** UMAP embedding fidelity. **(B)** t-SNE embedding fidelity. **(C)** Latent manifold structure. **(D)** Intrinsic quality and robustness.

Despite the graph-based models’ design for local structure, LiVAE proved highly competitive, significantly outperforming CLEAR (Δ=0.324) , scGNN (Δ=0.082) , and scGCC (Δ=0.048) in UMAP local quality (all p<0.001 ; Figure 5A). LiVAE established commanding leads in global embedding fidelity, with UMAP distance correlation improvements of Δ=0.398 to Δ=0.491 , yielding overall UMAP quality gains of Δ=0.208 to Δ=0.338 . The t-SNE results (Figure 5B) paralleled these findings ( Δ=0.176 to Δ=0.375 for overall quality).

Model stability was exceptional, with noise resilience substantially higher than that of all comparators ( Δ=0.545 to Δ=0.722 , all p<0.001 ; Figure 5D). Manifold complexity (Figure 5C) surpassed CLEAR across all metrics; comparisons with scGCC for dimensionality (Δ=0.281) and the participation ratio (Δ=0.348) showed numerical advantages not reaching statistical significance. While scGNN produced higher anisotropy (Δ=−0.146) , LiVAE produced superior overall intrinsic quality ( Δ=0.197 to Δ=0.495 ; Figure 5D), demonstrating that Lorentz-regularized information bottlenecks provide a powerful alternative to graph-based regularization.

### 3.5 Versatile and robust performance on chromatin accessibility data

We evaluated LiVAE on scATAC-seq data against three specialized methods (LSI, PeakVI, and PoissonVI) across n=82 datasets, demonstrating its cross-modality versatility and unique strengths in capturing the global structure and trajectory information (Table 5).

**TABLE 5:** Performance differences between LiVAE and scATAC-seq-specialized methods across n=82 scATAC-seq datasets. Values represent absolute differences ( Δ ; LiVAE − baseline). Significance levels: ^***^ p<0.001 , ^**^ p<0.01 , and ^*^ p<0.05 .

Metric	vs. LSI	vs. PeakVI	vs. PoissonVI
*UMAP embedding fidelity*			
*Dist. corr*	$0.082^{***}$	$0.080^{***}$	$0.155^{***}$
*Q local*	$0.066^{***}$	$0.078^{***}$	$0.062^{***}$
*Q global*	$0.052^{***}$	$0.059^{***}$	$0.084^{***}$
*Overall qual*	$0.067^{***}$	$0.072^{***}$	$0.100^{***}$
*t-SNE embedding fidelity*			
*Dist. corr*	$0.093^{***}$	$0.089^{***}$	$0.151^{***}$
*Q local*	$0.060^{***}$	$0.086^{***}$	$0.057^{***}$
*Q global*	$0.054^{***}$	$0.078^{***}$	$0.093^{***}$
*Overall qual*	$0.069^{***}$	$0.085^{***}$	$0.100^{***}$
*Latent manifold structure*			
*Manif. dim*	$0.133^{***}$	$0.170^{***}$	$0.191^{***}$
*Spectral decay*	$0.079^{***}$	$0.103^{***}$	$0.115^{***}$
*Part. ratio*	$0.142^{***}$	$0.250^{***}$	$0.303^{***}$
*Anisotropy*	$0.151^{***}$	$0.161^{***}$	$0.089^{***}$
*Intrinsic quality and robustness*			
*Core intrin. qual*	$0.126^{***}$	$0.171^{***}$	$0.174^{***}$
*Traj. directionality*	$0.138^{***}$	$0.225^{***}$	$0.321^{***}$
*Noise resilience*	$0.311^{***}$	$0.354^{***}$	$0.404^{***}$
*Overall intrin. qual*	$0.167^{***}$	$0.224^{***}$	$0.264^{***}$

**FIGURE 6:**
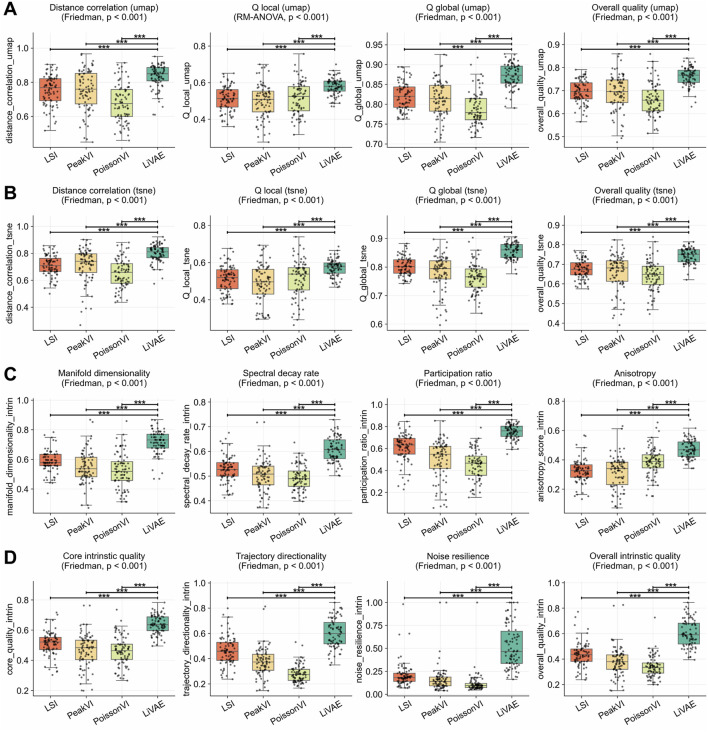
Comparison with scATAC-seq-specialized methods. Boxplots compare LiVAE with LSI, PeakVI, and PoissonVI across n=82 scATAC-seq datasets. Boxes indicate the median and IQR; whiskers extend to 1.5× IQR. Statistical significance was assessed using Bonferroni-corrected Wilcoxon tests (α=0.0083) . **(A)** UMAP embedding fidelity. **(B)** t-SNE embedding fidelity. **(C)** Latent manifold structure. **(D)** Intrinsic quality and robustness.

Against LSI and PeakVI, LiVAE was unequivocally superior across all categories. Overall intrinsic quality (Figure 6D) improved by Δ=0.167 and Δ=0.224 , driven by noise resilience gains of Δ=0.311 and Δ=0.354 , confirming robustness to sparse chromatin data. Manifold structure (Figure 6C) exceeded both methods across all four metrics: dimensionality (Δ=0.133/0.170) , spectral decay (Δ=0.079/0.103) , participation ratio (Δ=0.142/0.250) , and anisotropy ( Δ=0.151/0.161 , all p<0.001 ).

The comparison against PoissonVI revealed LiVAE’s complementary strengths. LiVAE achieved higher core intrinsic quality (Δ=0.174) and dramatically superior trajectory directionality ( Δ=0.321 ; Figure 6D), suggesting better capture of continuous biological processes. Its geometric regularization provided superior global structure (UMAP distance correlation Δ=0.155 ; Figure 6A) and overall intrinsic quality (Δ=0.264) , alongside exceptional noise resilience ( Δ=0.404 , all p<0.001 ). These results confirm LiVAE as a powerful tool for scATAC-seq analysis, offering compelling advantages for studies prioritizing global landscape understanding and developmental trajectory inference.

### 3.6 Dual loss pathways provide complementary representational benefits

We performed ablation studies on n=53 scRNA-seq datasets, systematically removing either the main reconstruction or the bottleneck pathway under both BN and Lorentz-regularized configurations. The two pathways perform highly specialized, non-redundant functions, with the full model decisively outperforming any simplified variant (Table 6).

**TABLE 6:** Performance differences for dual-pathway ablations under bottleneck-only (BN) and Lorentz-regularized (Lorentz) configurations across n=53 scRNA-seq datasets. Values represent absolute differences ( Δ ; full model − ablation). All differences are derived from paired comparisons; formal significance testing was not applied.

Metric	BN architecture		Lorentz architecture	
	vs. w/o main	vs. w/o BN	vs. w/o main	vs. w/o BN
*Clustering performance*				
*NMI*	0.122	0.009	0.121	0.015
*ARI*	0.123	0.009	0.121	0.015
*ASW*	0.142	0.062	0.129	0.062
*DAV*	−0.612	−0.286	−0.520	−0.310
*CAL*	724.352	729.417	635.618	712.973
*COR*	0.873	1.434	0.786	1.339
*UMAP embedding fidelity*				
*Dist. corr*	0.060	0.198	0.037	0.208
*Q local*	0.158	0.070	0.146	0.071
*Q global*	0.031	0.077	0.025	0.079
*Overall qual*	0.083	0.115	0.069	0.119
*t-SNE embedding fidelity*				
*Dist. corr*	0.088	0.223	0.052	0.227
*Q local*	0.189	0.068	0.181	0.070
*Q global*	0.069	0.088	0.053	0.089
*Overall qual*	0.115	0.126	0.095	0.129
*Latent manifold structure*				
*Manif. dim*	0.069	0.234	0.047	0.235
*Spectral decay*	0.017	0.095	−0.001	0.095
*Part. ratio*	0.106	0.355	0.057	0.356
*Anisotropy*	−0.047	0.151	−0.065	0.156
*Intrinsic quality and robustness*				
*Core intrin. qual*	0.037	0.209	0.010	0.210
*Traj. directionality*	0.063	0.178	0.032	0.177
*Noise resilience*	0.050	0.141	0.020	0.132
*Overall intrin. qual*	0.047	0.186	0.018	0.185

**FIGURE 7:**
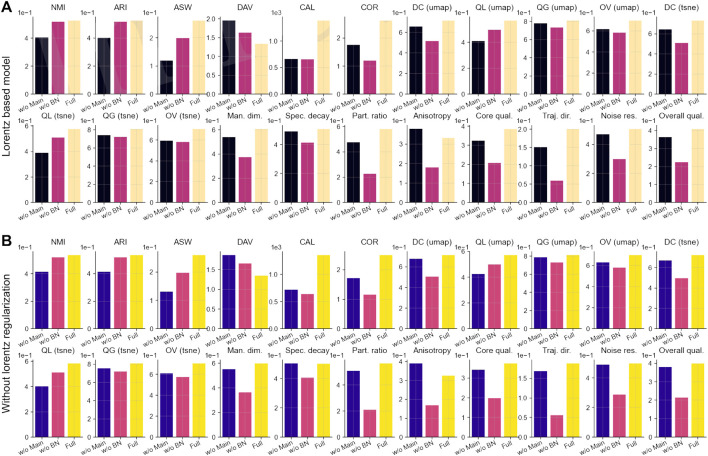
Ablation analysis of dual loss pathways. **(A)** Bottleneck-only configuration (BN): full model vs. w/o main and w/o BN, n=53 scRNA-seq datasets. **(B)** Lorentz-regularized configuration (Lorentz): full model vs. w/o main and w/o BN, n=53 scRNA-seq datasets.

Removing the bottleneck pathway (“w/o BN”) caused catastrophic collapse in geometric integrity. Overall intrinsic quality increased by Δ=0.186 (BN; Figure 7A) and Δ=0.185 (Lorentz; Figure 7B), while the participation ratio decreased by Δ=0.355/0.356 . UMAP distance correlation declined by Δ=0.198/0.208 , with overall quality decreasing by Δ=0.115/0.119 . Remarkably, supervised clustering (NMI/ARI) declined minimally (Δ≈0.01) , demonstrating the bottleneck’s primary role in establishing geometric robustness rather than defining discrete clusters.

Conversely, removing the main pathway (“w/o main”) produced an inverted deficiency profile. Supervised clustering accuracy collapsed (NMI/ARI Δ≈0.12 ; CAL Δ=635 –724), while intrinsic quality (Δ=0.018/0.047) and manifold structure worsened less severely. This indicates that the main pathway refines latent space for categorical label separation, operating upon the bottleneck’s stable geometric foundation. These complementary roles prove that both pathways are indispensable for state-of-the-art performance.

### 3.7 A deterministic anchor point fortifies geometric regularization

**FIGURE 8:**
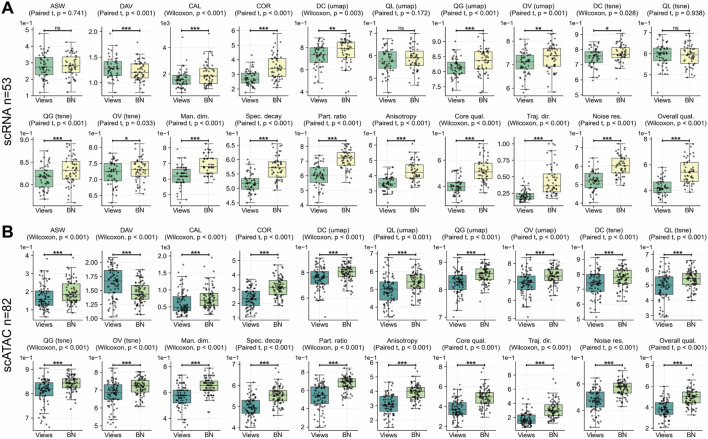
Comparison of Lorentz-regularization strategies. **(A)** scRNA-seq (n=53) : bottleneck-anchored (BN) vs. two-view sampled (Views). **(B)** scATAC-seq (n=82) : BN vs. Views.

To establish the most effective method for applying Lorentz-distance regularization, we contrasted two strategies: anchoring the calculation to the deterministic information BN versus using two independently sampled posterior views (Views). Evaluation on scRNA-seq (n=53) and scATAC-seq (n=82) datasets revealed that the bottleneck-anchored strategy was superior across all metric categories (Figures 8A,B; Table 7).

**TABLE 7:** Performance differences between bottleneck-anchored (BN) and two-view sampled (Views) Lorentz regularization across scRNA-seq (n=53) and scATAC-seq (n=82) datasets. Values represent absolute differences ( Δ ; BN − Views). Significance levels: ^***^ p<0.001 , ^**^ p<0.01 , and ^*^ p<0.05 .

Metric	scRNA-seq	scATAC-seq
*Clustering performance*		
*ASW*	0.002	$0.023^{***}$
*DAV*	$-0.072^{***}$	$-0.168^{***}$
*CAL*	$405.759^{***}$	$164.000^{***}$
*COR*	$0.736^{***}$	$0.743^{***}$
*UMAP embedding fidelity*		
*Dist. corr*	$0.046^{**}$	$0.046^{***}$
*Q local*	0.007	$0.045^{***}$
*Q global*	$0.021^{***}$	$0.029^{***}$
*Overall qual*	$0.025^{**}$	$0.040^{***}$
*t-SNE embedding fidelity*		
*Dist. corr*	$0.023^{*}$	$0.038^{***}$
*Q local*	−0.000	$0.047^{***}$
*Q global*	$0.015^{***}$	$0.033^{***}$
*Overall qual*	$0.013^{*}$	$0.039^{***}$
*Latent manifold structure*		
*Manif. dim*	$0.075^{***}$	$0.075^{***}$
*Spectral decay*	$0.052^{***}$	$0.055^{***}$
*Part. ratio*	$0.109^{***}$	$0.142^{***}$
*Anisotropy*	$0.081^{***}$	$0.087^{***}$
*Intrinsic quality and robustness*		
*Core intrin. qual*	$0.079^{***}$	$0.090^{***}$
*Traj. directionality*	$0.118^{***}$	$0.116^{***}$
*Noise resilience*	$0.225^{***}$	$0.140^{***}$
*Overall intrin. qual*	$0.120^{***}$	$0.108^{***}$

The BN approach’s most profound impact was on model robustness and manifold quality. Noise resilience increased dramatically ( Δ=0.225 for scRNA-seq; Δ=0.140 for scATAC-seq), driving overall intrinsic quality gains of Δ=0.120 and Δ=0.108 , respectively. This stability was mirrored in latent geometry: participation ratio improved by Δ=0.109 and Δ=0.142 , respectively; manifold dimensionality improved by Δ=0.075 for both modalities; and trajectory directionality improved by Δ=0.118 and Δ=0.116 , respectively. These structural gains translated to better embedding fidelity (UMAP overall quality: Δ=0.025 and Δ=0.040 ; t-SNE: Δ=0.013 and Δ=0.039 ), improved cluster compactness (CAL: Δ=405.759 and Δ=164.000 ), and enhanced latent dimension coupling (COR: Δ=0.736 and Δ=0.743 ), indicating stronger preservation of coordinated biological programs. The average silhouette width showed minimal change for scRNA-seq (Δ=0.002) but notable improvement for scATAC-seq (Δ=0.023) .

These results indicate that anchoring Lorentz regularization to a fixed, deterministic reference point mitigates training instability inherent in stochastic sampling. The bottleneck provides a consistent geometric scaffold, enabling more coherent latent organization across all analysis modalities.

### 3.8 Optimizing data fidelity through modality-aware reconstruction

**FIGURE 9:**
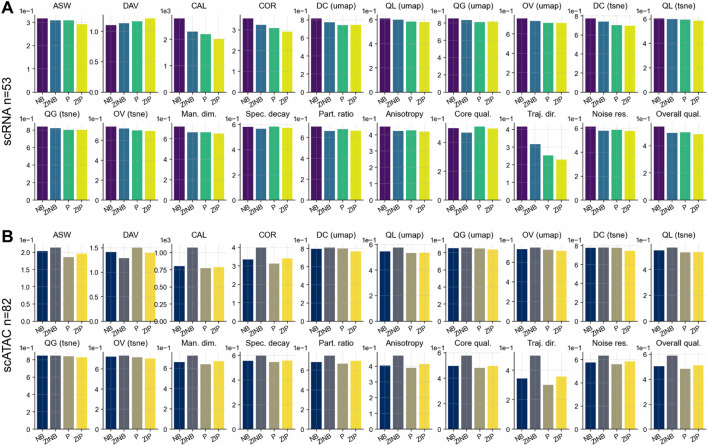
Evaluation of reconstruction likelihood functions. **(A)** scRNA-seq (n=53) : LiVAE with NB, ZINB, Poisson, and ZI-Poisson. **(B)** scATAC-seq (n=82) : LiVAE with NB, ZINB, Poisson, and ZI-Poisson.

Selecting an appropriate reconstruction loss is crucial for modeling distinct single-cell assay properties. We benchmarked four likelihoods—NB, ZINB, Poisson, and ZI-Poisson—on scRNA-seq (n=53) and scATAC-seq (n=82) datasets (Figures 9A,B; Table 8).

**TABLE 8:** Performance differences for reconstruction likelihood functions across scRNA-seq (n=53) and scATAC-seq (n=82) datasets. Values represent absolute differences ( Δ ; optimal loss − alternative). For scRNA-seq: NB vs. others; for scATAC-seq: ZINB vs. others. All differences are derived from paired comparisons; formal significance testing was not applied.

Metric	scRNA-seq (NB-based)			scATAC-seq (ZINB-based)		
	vs. ZINB	vs. Poisson	vs. ZI-Poisson	vs. NB	vs. Poisson	vs. ZI-Poisson
*Clustering performance*						
*ASW*	0.007	0.008	0.024	0.010	0.028	0.017
*DAV*	−0.031	−0.063	−0.109	−0.124	−0.214	−0.113
*CAL*	488.822	597.291	768.543	268.195	300.059	285.634
*COR*	0.305	0.454	0.624	0.632	0.861	0.588
*UMAP embedding fidelity*						
*Dist. corr*	0.043	0.074	0.071	0.012	0.011	0.040
*Q local*	0.011	0.025	0.029	0.029	0.042	0.040
*Q global*	0.019	0.038	0.037	0.005	0.008	0.020
*Overall qual*	0.024	0.046	0.046	0.015	0.020	0.033
*t-SNE embedding fidelity*						
*Dist. corr*	0.034	0.071	0.076	0.003	0.003	0.034
*Q local*	0.004	0.011	0.018	0.021	0.037	0.035
*Q global*	0.017	0.035	0.036	0.001	0.008	0.018
*Overall qual*	0.018	0.039	0.044	0.008	0.016	0.029
*Latent manifold structure*						
*Manif. dim*	0.055	0.057	0.068	0.064	0.084	0.053
*Spectral decay*	0.014	−0.003	0.006	0.042	0.051	0.038
*Part. ratio*	0.043	0.023	0.039	0.063	0.082	0.052
*Anisotropy*	0.027	0.024	0.030	0.063	0.076	0.052
*Intrinsic quality and robustness*						
*Core intrin. qual*	0.035	0.025	0.036	0.058	0.073	0.049
*Traj. directionality*	0.032	−0.010	0.004	0.079	0.096	0.080
*Noise resilience*	0.100	0.162	0.186	0.154	0.196	0.140
*Overall intrin. qual*	0.047	0.042	0.056	0.084	0.105	0.076

For scRNA-seq, NB provided the most robust performance (Figure 9A). Its primary advantage was enhanced noise resilience ( Δ=0.100 to Δ=0.186 vs. alternatives) and overall intrinsic quality ( Δ=0.047 to Δ=0.056 ), translating to superior cluster compactness (CAL: Δ up to 768.543) and stronger latent dimension coupling (COR: Δ up to 0.624), reflecting better preservation of interdependent biological programs. Embedding fidelity showed consistent but modest improvements (UMAP overall quality: Δ=0.024 to Δ=0.046 ). While Poisson achieved marginal advantages in trajectory directionality (Δ=−0.010) and spectral decay (Δ=−0.003) , NB’s robustness benefits outweighed these task-specific trade-offs. The NB distribution effectively models the overdispersion characteristic of gene expression without requiring explicit zero-inflation handling.

For scATAC-seq, ZINB was superior (Figure 9B), driven by better noise resilience ( Δ up to 0.196) and manifold structure (participation ratio: Δ=0.063 vs. NB). ZINB achieved stronger latent dimension coupling (COR: Δ=0.632 vs. NB), indicating better preservation of coordinated regulatory programs and higher overall intrinsic quality ( Δ=0.084 vs. NB, Δ=0.105 vs. Poisson). This advantage reflects ZINB’s ability to explicitly model both overdispersion and the extreme zero-inflation inherent in sparse chromatin accessibility data, making it essential for accurate scATAC-seq representation.

### 3.9 Robustness and hyperparameter stability

**FIGURE 10:**
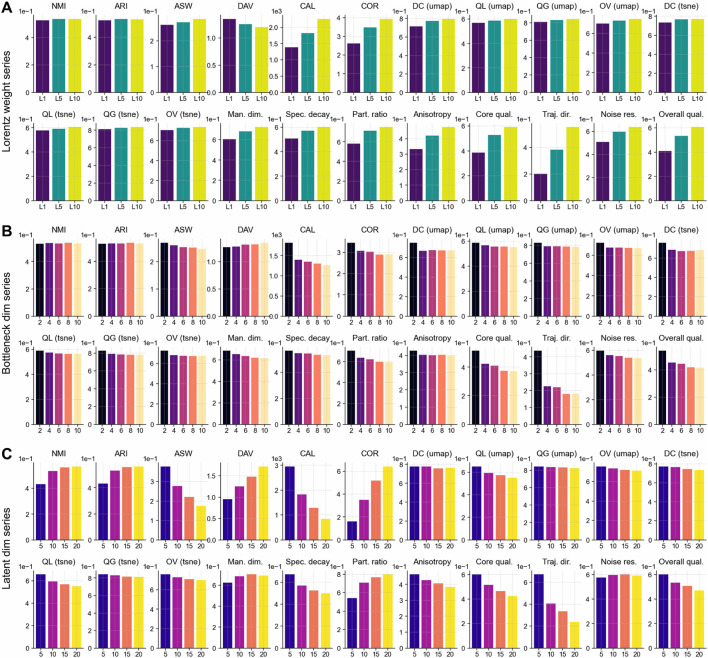
Hyperparameter sensitivity analysis. **(A)** Lorentz-regularization weight (λ∈{1,5,10}) on n=53 scRNA-seq datasets. **(B)** Information bottleneck dimensionality $\left(d_{\text{BN}}\in \left\{2,4,6,8,10\right\}\right)$ on n=53 scRNA-seq datasets. **(C)** Latent dimensionality $\left(d_{\text{latent}}\in \left\{5,10,15,20\right\}\right)$ on n=53 scRNA-seq datasets.

We evaluated LiVAE’s sensitivity to key hyperparameters on n=53 scRNA-seq datasets: Lorentz-regularization weight (λ∈{1,5,10}) , bottleneck dimensionality $\left(d_{\text{BN}}\in \left\{2,4,6,8,10\right\}\right)$ , and latent dimensionality $\left(d_{\text{latent}}\in \left\{5,10,15,20\right\}\right)$ , which are visualized in Figures 10A–C and quantified in Table 9.

**TABLE 9:** Performance differences for hyperparameter ablations across n=53 scRNA-seq datasets. Values represent absolute differences ( Δ ; optimal setting − alternative). Lorentz weight: λ=10 vs. others; bottleneck dim.: $d_{\text{BN}}=2$ vs. others; latent dim.: $d_{\text{latent}}=10$ vs. others. All differences are derived from paired comparisons; formal significance testing was not applied.

Metric	Lorentz weight		Bottleneck dim				Latent dim		
	vs. L1	vs. L5	vs. 4	vs. 6	vs. 8	vs. 10	vs. 5	vs. 15	vs. 20
*Clustering performance*									
*NMI*	0.007	−0.002	−0.004	−0.004	−0.009	−0.003	0.101	−0.028	−0.037
*ARI*	0.007	−0.002	−0.004	−0.004	−0.009	−0.003	0.100	−0.026	−0.033
*ASW*	0.023	0.013	0.009	0.018	0.018	0.023	−0.102	0.056	0.101
*DAV*	−0.149	−0.053	−0.016	−0.047	−0.058	−0.088	0.299	−0.220	−0.467
*CAL*	871.749	436.057	423.691	472.544	524.646	559.683	−1134.477	533.832	969.863
*COR*	1.311	0.459	0.383	0.428	0.570	0.555	1.863	−1.699	−2.942
*UMAP embedding fidelity*									
*Dist. corr*	0.080	0.024	0.085	0.077	0.081	0.079	0.003	0.021	0.016
*Q local*	0.032	0.014	0.022	0.031	0.032	0.036	−0.059	0.019	0.043
*Q global*	0.034	0.011	0.041	0.041	0.044	0.047	−0.008	0.005	0.011
*Overall qual*	0.049	0.016	0.049	0.049	0.052	0.054	−0.022	0.015	0.023
*t-SNE embedding fidelity*									
*Dist. corr*	0.039	0.006	0.076	0.087	0.084	0.078	−0.009	0.021	0.030
*Q local*	0.027	0.014	0.014	0.021	0.025	0.025	−0.062	0.025	0.042
*Q global*	0.025	0.008	0.035	0.043	0.044	0.044	−0.011	0.013	0.016
*Overall qual*	0.030	0.009	0.042	0.050	0.051	0.049	−0.027	0.020	0.029
*Latent manifold structure*									
*Manif. dim*	0.118	0.043	0.033	0.048	0.066	0.067	0.059	−0.018	−0.006
*Spectral decay*	0.093	0.030	0.020	0.021	0.032	0.034	−0.102	0.042	0.069
*Part. ratio*	0.168	0.039	0.065	0.082	0.107	0.110	0.166	−0.061	−0.090
*Anisotropy*	0.143	0.054	0.024	0.025	0.024	0.029	−0.036	0.019	0.042
*Intrinsic quality and robustness*									
*Core intrin. qual*	0.131	0.042	0.035	0.044	0.057	0.060	0.022	−0.005	0.004
*Traj. directionality*	0.205	0.063	0.090	0.104	0.139	0.143	−0.085	0.050	0.089
*Noise resilience*	0.353	0.171	0.211	0.216	0.256	0.255	−0.265	0.070	0.167
*Overall intrin. qual*	0.197	0.074	0.087	0.096	0.122	0.124	−0.068	0.027	0.062

Increasing λ from 1 to 10 consistently improved performance (Figure 10A), particularly overall intrinsic quality (Δ=0.197) , driven by gains in noise resilience (Δ=0.353) , trajectory directionality (Δ=0.205) , and the participation ratio (Δ=0.168) . Clustering compactness (CAL: Δ=871.749 ) and embedding fidelity (UMAP overall quality: Δ=0.049 ) also improved. Moving from λ=5 to λ=10 showed diminishing but positive returns across most metrics, suggesting that stronger regularization is generally beneficial.

The bottleneck dimensionality analysis revealed that $d_{\text{BN}}=2$ achieved optimal performance in unsupervised metrics (Figure 10B). Compared to $d_{\text{BN}}=10$ , it improved clustering compactness (CAL: Δ=559.683 ), embedding quality (UMAP: Δ=0.054 ; t-SNE: Δ=0.049 ), and manifold structure (participation ratio: Δ=0.110 ), with minimal impact on supervised clustering (NMI: Δ=−0.003 ). This tight bottleneck effectively isolates core biological signals while filtering technical noise.

Latent dimensionality presented a clear trade-off (Figure 10C). The $d_{\text{latent}}=10$ setting balanced supervised clustering accuracy (NMI: Δ=0.101 vs. d=5 ; Δ=−0.037 vs. d=20 ) with unsupervised compactness. Lower dimensions (d=5) underfit the complex structure, while higher dimensions (d=20) reduced cluster compactness (CAL: Δ=969.863 worse than d=10 ) and weakened latent dimension coupling (COR: Δ=−2.942 ), suggesting diminished preservation of coordinated biological programs.

These findings support the default settings of λ=10 , $d_{\text{BN}}=2$ , and $d_{\text{latent}}=10$ for typical analyses, with $d_{\text{latent}}$ adjustable based on the dataset complexity.

### 3.10 Emergent batch correction and robust clustering performance

LiVAE’s information bottleneck and geometric regularization promote globally coherent embeddings that can disentangle biological signals from batch effects without explicit batch-correction terms. We benchmarked multi-batch scRNA-seq integration against scVI, scDHMap, scDeepCluster, scGNN, and scGCC on 21 multi-batch datasets.

**FIGURE 11:**
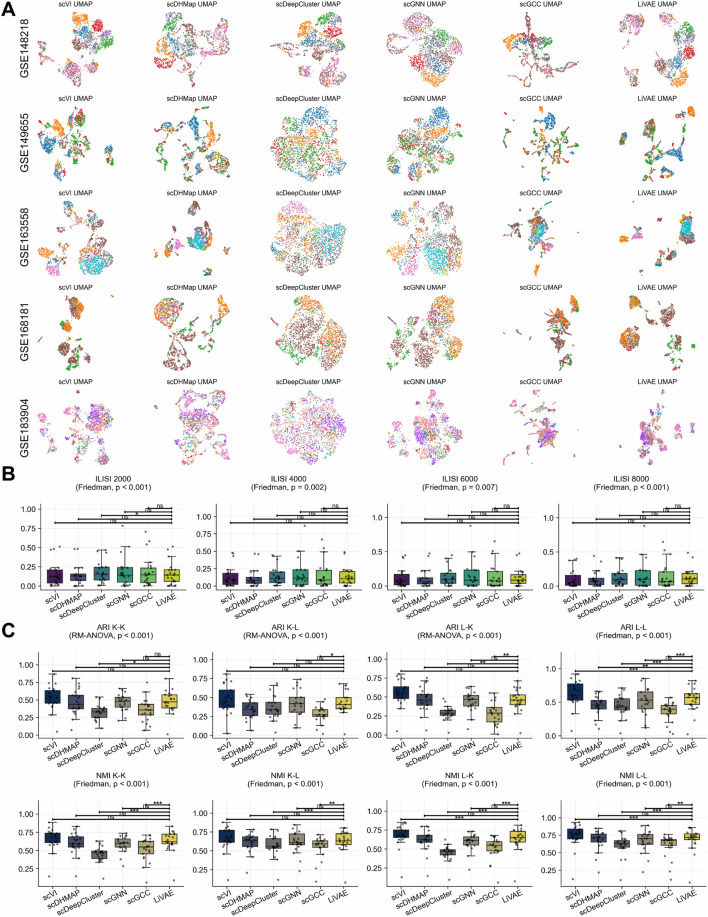
Batch integration and supervised clustering across multi-batch scRNA-seq datasets. **(A)** Representative UMAP embeddings from LiVAE and five comparison methods across five multi-batch datasets, colored by batch. **(B)** iLISI evaluation across downsampled cell counts (2,000–8,000); LiVAE achieves comparable batch mixing to specialized methods across n=21 datasets. **(C)** Supervised clustering accuracy (ARI and NMI) using four combinations of pre- and post-clustering algorithms: K-means/K-means (K–K), K-means/Leiden (K–L), Leiden/K-means (L–K), and Leiden/Leiden (L–L).

UMAP visualizations of five representative datasets show well-mixed embeddings preserving biological structure (Figure 11A). Quantitative iLISI evaluation across the full set of 21 datasets with 2,000–8,000 cell subsamplings revealed that LiVAE achieves batch mixing comparable to that with specialized methods (Figure 11B).

Supervised clustering evaluation using four pipelines—combining K-means or Leiden for pre- and post-integration clustering (denoted as K–K, K–L, L–K, and L–L)—showed mixed results (Figure 11C; Table 10). LiVAE substantially outperformed scDeepCluster (ARI: Δ=0.101 to Δ=0.167 ) and scGCC ( Δ=0.117 to Δ=0.180 ) across most pipelines. However, scVI achieved superior accuracy in Leiden-based strategies (ARI: Δ=−0.091 to Δ=−0.095 ; NMI: Δ=−0.041 to Δ=−0.051 ).

**TABLE 10:** Paired differences in supervised clustering across four clustering strategies evaluated on multi-batch single-cell datasets ( n=21 experimental conditions from five datasets). Values represent absolute differences ( Δ ; LiVAE − comparator). Significance levels: ^*^ p<0.05 , ^**^ p<0.01 , and ^***^ p<0.001 . Negative values with markers indicate significant superiority of the comparator; positive values with markers indicate significant superiority of LiVAE. Absence of markers indicates non-significant differences.

Metric	vs. scVI	vs. scDHMap	vs. scDeepCluster	vs. scGNN	vs. scGCC
*ARI*					
*K-means/K-means*	−0.054	0.009	$0.157^{*}$	0.011	0.117
*K-means/Leiden*	−0.044	0.090	0.054	0.011	$0.154^{*}$
*Leiden/K-means*	−0.091	−0.008	$0.167^{**}$	0.016	$0.168^{**}$
*Leiden/Leiden*	$-0.095^{***}$	$0.089^{**}$	$0.101^{***}$	0.026	$0.180^{***}$
*NMI*					
*K-means/K-means*	−0.028	0.026	$0.180^{***}$	0.041	$0.112^{***}$
*K-means/Leiden*	−0.020	0.020	$0.065^{***}$	0.016	$0.078^{**}$
*Leiden/K-means*	$-0.041^{***}$	0.017	$0.183^{***}$	0.042	$0.119^{***}$
*Leiden/Leiden*	$-0.051^{***}$	0.020	$0.087^{***}$	0.024	$0.093^{**}$

These findings demonstrate that LiVAE provides competitive batch integration and stable clustering without specialized batch parameters. Although dedicated batch-correction methods may be preferred for datasets with extreme confounding, LiVAE offers a versatile, general-purpose solution for integrated single-cell analysis.

### 3.11 Biological interpretability of latent components on a *Dapp1* perturbation dataset

**FIGURE 12:**
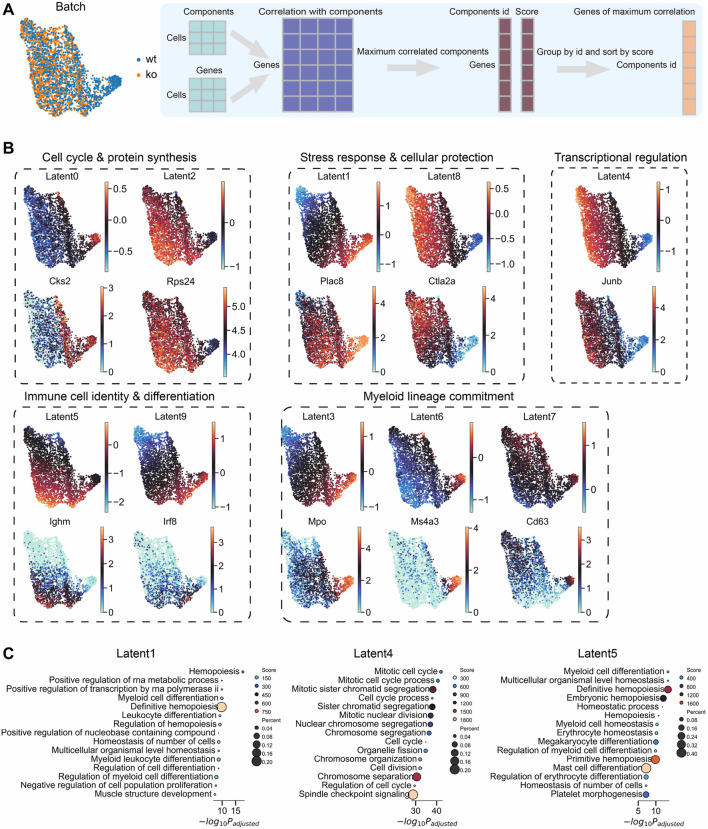
Interpretability of LiVAE latent components in a Dapp1 perturbation scRNA-seq dataset. **(A)** UMAP of cells from GSE277292, colored by condition (wt: wild-type; ko: knockout), and schematic of the gene-component association workflow based on the maximum expression correlation. **(B)** UMAPs of selected component activation scores (top of each pair) alongside expression of the most correlated marker genes (bottom). Functional groupings include cell cycle and protein synthesis (Latent0/*Cks2* and Latent2/*Rps24*); stress response and cellular protection (Latent1/*Plac8* and Latent8/*Ctla2a*); transcriptional regulation (Latent4/*Junb*); immune identity and differentiation (Latent5/*Ighm* and Latent9/*Irf8*); and myeloid lineage commitment (Latent3/*Mpo*, Latent6/*Ms4a3*, and Latent7/*Cd63*). **(C)** Gene Ontology biological process (GOBP) enrichment for the top correlated genes with Latent1, Latent4, and Latent5. Dot size indicates the gene count; color encodes adjusted p -value. Results support roles in hemopoiesis, mitotic cell-cycle processes, and myeloid differentiation.

To assess LiVAE’s biological interpretability, we analyzed hematopoietic stem and progenitor cell scRNA-seq with *Dapp1* knockout perturbation (GSE277292). UMAP embedding showed consistent cellular structure with minimal batch effects between wild-type and knockout conditions (Figure 12A). We annotated components by identifying genes with the highest per-cell expression–activation correlation.

Individual components captured distinct biological programs (Figure 12B). Cell-cycle and protein synthesis were tracked by Latent0 (*Cks2*) and Latent2 (*Rps24*). Stress-response programs aligned with Latent1 [*Plac8* (Rogulski et al., 2005)] and Latent8 (*Ctla2a*). Transcriptional regulation was reflected in Latent4 [*Junb* (Santilli et al., 2021)]. Immune identity was found through Latent5 [*Ighm* (Dobre et al., 2021)] and Latent9 [*Irf8* (Kurotaki and Tamura, 2019)], while myeloid commitment was found through Latent3 [*Mpo* (Lanza et al., 2001)], Latent6 [*Ms4a3* (Donato et al., 2002)], and Latent7 [*Cd63* (Pols and Klumperman, 2009)].

Gene Ontology biological process enrichment corroborated these assignments (Figure 12C): Latent4 enriched for “mitotic cell cycle,” Latent1 enriched for “hemopoiesis,” and Latent5 enriched for “myeloid differentiation.” These results demonstrate that LiVAE decomposes transcriptomes into disentangled, biologically meaningful axes, facilitating data-driven hypothesis generation.

## 4 Discussion

We introduced LiVAE, a geometrically regularized variational autoencoder that address the local–global trade-off in single-cell representation learning. Through systematic benchmarking across 135 datasets against 21 baseline methods spanning classical dimensionality reduction, deep generative models, graph-based architectures, and modality-specific approaches, we demonstrated that LiVAE achieves higher global topology preservation, richer latent manifold geometry, and enhanced robustness while maintaining competitive local structure fidelity. These technical advances translate to improved biological discovery: LiVAE embeddings better preserve developmental hierarchies, enable more accurate cell-type annotation, and provide interpretable latent dimensions aligned with known biological processes.

Unlike prior hyperbolic deep learning approaches that constrain entire latent spaces to hyperbolic manifolds (Park et al., 2021)—requiring manifold-aware operations, hyperbolic priors, and specialized reparameterization that increase computational cost and reduce flexibility (Cho et al., 2023)—LiVAE applies hyperbolic geometry only as regularization over a standard Euclidean latent space $z\in R^{d}$ . This hybrid design offers three key advantages with direct biological utility: downstream compatibility ( z works seamlessly with standard clustering, trajectory inference, and visualization tools), noise filtering (the dimensional bottleneck $d_{c}\ll d$ discards batch effects and technical noise while retaining hierarchical biological structure), and architectural decoupling (separate pathways optimize reconstruction fidelity and geometric structure, balancing local accuracy with global coherence). Our ablation studies empirically validate this design: the main pathway primarily controls the categorical structure (NMI and ARI), while the bottleneck pathway governs geometric quality (distance correlation and participation ratio) and robustness, with deterministic $l_{e}$ providing stable geometric regularization across training iterations.

We use the full latent vector z (d=10) rather than the compressed $l_{e}$ $\left(d_{c}=2\right)$ because these serve distinct roles: $l_{e}$ distills minimal global structure for geometric regularization, while z retains the capacity for both global topology and local variation needed for clustering, marker identification, and trajectory inference (Ding et al., 2018). Our sensitivity analysis confirms that d=10 optimally balances performance and interpretability, enabling decomposition into biologically interpretable components that would be lost at lower dimensions. For scATAC-seq data, which exhibit substantially higher zero rates than scRNA-seq due to biological sparsity and technical dropout (Yan et al., 2020), we adopt ZINB reconstruction loss. Empirical evaluation confirms that ZINB consistently outperforms NB, Poisson, and ZIP on scATAC-seq datasets, yielding 47% relative improvement in latent dimension coupling (COR: Δ=0.632 vs. NB), indicating stronger preservation of coordinated regulatory programs, alongside gains in trajectory directionality (Δ=0.079) and noise resilience (Δ=0.154) , aligning with recent ZINB-based scATAC-seq frameworks (Lan et al., 2023; Rachid Zaim et al., 2024).

While not explicitly designed for batch correction, LiVAE achieves comparable iLISI scores to scVI across 21 multi-batch datasets through three mechanisms: the information bottleneck attenuates batch-specific artifacts orthogonal to biological signal (Voloshynovskiy et al., 2019), geometric loss enforces global coherence that implicitly aligns cross-batch representations, and shared decoders incentivize batch-invariant features. For datasets with severe batch confounding (e.g., cell types appearing in only one batch), scVI’s explicit batch modeling may be superior, but LiVAE’s simpler architecture—requiring no batch labels and avoiding adversarial training instabilities—offers practical advantages for routine integration.

Based on our systematic benchmarking, we recommend using LiVAE when the dataset structure is unknown and exploratory analysis is needed, global topology preservation is critical (e.g., identifying rare populations and inferring developmental hierarchies), or cross-dataset integration is required without batch labels. Alternative methods should be used when the trajectory structure is well-defined and pseudotime accuracy is paramount (prefer scDHMap and scTour), extreme sparsity ( >98% zeros) dominates scATAC-seq (consider PoissonVI), or supervised batch correction with known batch identities is available (scVI offers marginal advantages in highly confounded scenarios).

Several limitations motivate future development. First, while our component-wise interpretability analysis demonstrates that latent dimensions capture biologically meaningful variation, LiVAE does not enforce strict disentanglement—the components may exhibit residual correlations, unlike β -VAE or FactorVAE frameworks that explicitly penalize dependencies (Burgess et al., 2018; Kim et al., 2018). Extending to true causal disentanglement (Sikka et al., 2019; Abdelaleem et al., 2025) would enable more principled perturbation analysis. Second, LiVAE does not currently handle paired multi-omic measurements (e.g., 10x multiome scRNA + scATAC from the same cells); extending to true multi-modal integration would require modality-specific encoders, cross-modal alignment losses, and validation on datasets such as SHARE-seq or 10x multiome (Zuo and Chen, 2021). Third, experimental validation—such as comparing LiVAE-guided cell sorting with ground-truth lineage tracing—would strengthen claims of biological relevance but requires specialized datasets that are currently unavailable for most benchmarked tissues.

Beyond these immediate needs, our results establish geometric regularization—specifically Lorentzian distance constraints across information bottlenecks—as a powerful strategy for learning hierarchical representations that extend beyond transcriptomics to spatial transcriptomics (cells → microenvironments → tissue regions), protein interaction networks, and metabolic pathways with tree-like structure (Pogány et al., 2024; Li et al., 2023). Adaptive curvature learning (Skopek et al., 2020) would enable automatic tuning of geometric constraints to dataset-specific hierarchies. In conclusion, LiVAE establishes geometric regularization as a practical alternative to graph-based and batch-correction-centric approaches in single-cell representation learning, achieving state-of-the-art global topology preservation, noise resilience, and interpretability without sacrificing local fidelity. Our open-source implementation and comprehensive benchmarking framework enable community evaluation and extension, accelerating the integration of geometric deep learning into mainstream single-cell genomics workflows.

## Data Availability

The original contributions presented in the study are publicly available. The source code for this research is publicly available on GitHub at https://github.com/PeterPonyu/LiVAE. The single-cell sequencing data for the Dapp1 perturbation experiments is publicly available in the Gene Expression Omnibus (GEO) under accession number GSE277292.

## Glossary

ARI: Adjusted Rand index
BN: Bottleneck
CLEAR: Contrastive learning for scRNA-seq
DAV: Davies–Bouldin index
DIPVAE: Disentangled inferred prior VAE
FactorVAE: Factor variational autoencoder
HVGs: Highly variable genes
ICA: Independent component analysis
InfoVAE: Information maximizing VAE
KPCA: Kernel principal component analysis
LSI: Latent semantic indexing
NB: Negative binomial
NMI: Normalized mutual information
PCA: Principal component analysis
PoissonVI: Poisson variational inference
scATAC-seq: Single-cell ATAC sequencing
scDHMap: Single-cell deep hyperbolic manifold learning
scGNN: Single-cell graph neural network
scTour: Single-cell trajectory optimization by unsupervised representation
SDR: Spectral decay rate
TF-IDF: Term frequency-inverse document frequency
TSVD: Truncated singular value decomposition
VAE: Variational autoencoder
ZIP: Zero-inflated Poisson
β **-VAE**: β -variational autoencoder
ASW: Average silhouette width
CAL: Calinski–Harabasz index
COR: Coupling degree
DICL: Dictionary learning
FA: Factor analysis
HSD: Honest significant difference
HVPs: Highly variable peaks
iLISI: Integration local inverse Simpson’s index
KL: Kullback–Leibler
LiVAE: Lorentzian variational autoencoder
MD: Manifold dimensionality
NMF: Non-negative matrix factorization
NR: Noise resilience
PeakVI: Peak variational inference
PR: Participation ratio
scDeepCluster: Single-cell deep clustering
scGCC: Single-cell graph contrastive clustering
scRNA-seq: Single-cell RNA sequencing
scVI: Single-cell variational inference
TD: Trajectory directionality
t-SNE: t-distributed stochastic neighbor embedding
UMAP: Uniform manifold approximation and projection
ZINB: Zero-inflated negative binomial
β **-TCVAE**: Total correlation VAE with β weighting
