# Supplementary material for: Lorentz-regularized interpretable VAE for multi-scale single-cell transcriptomic and epigenomic embeddings
Source: Front Genet. 2026 Jan 5;16:1713727. doi: 10.3389/fgene.2025.1713727 (PMC12812404; doi:10.3389/fgene.2025.1713727)
Supplement: Supplementary file 1 [file Supplementaryfile1.pdf]

Supplementary File: Dataset Descriptions for Reproducibility

A comprehensive list of single-cell RNA sequencing and single-cell ATAC sequencing datasets retrieved from the Gene Expression Omnibus (GEO) database, including accession numbers, references, and relevant URLs for full reproducibility of the analysis.

A total of 53 single-cell RNA sequencing datasets were retrieved from the Gene Expression Omnibus (GEO) database for analysis.

- Sousa, C., Poovathingal, S. K., Kaoma, T., Azuaje, F., Skupin, A. and Michelucci, A. Single-cell transcriptomics reveals distinct microglia signatures under inflammation. GEO GSE115571. https://www.ncbi.nlm.nih.gov/geo/query/acc.cgi?acc=GSE115571
- Simon, L. M. and Schiller, H. B. Single cell RNA sequencing analysis of fresh resected human lung tissue. GEO GSE130148. https://www.ncbi.nlm.nih.gov/geo/query/acc.cgi?acc=GSE130148
- Zhang, S., Cui, Y., Wen, L., Qiao, J. and Tang, F. Single-cell transcriptomics reveals the divergent developmental lineage trajectories during human pituitary development. GEO GSE142653. https://www.ncbi.nlm.nih.gov/geo/query/acc.cgi?acc=GSE142653
- Joseph, D. B., et al. Single-cell RNA-sequencing of adult mouse lower urinary tracts. GEO GSE145929. https://www.ncbi.nlm.nih.gov/geo/query/acc.cgi?acc=GSE145929 *Provides two data samples
- Yuan, S., Liu, Q., Hu, Z. and Mao, X. Single-cell RNA-sequencing reveals the heterogeneity of microglia in fibrous membrane derived from proliferative diabetic retinopathy and proliferative vitreoretinopathy. GEO GSE165784. https://www.ncbi.nlm.nih.gov/geo/query/acc.cgi?acc=GSE165784
- Hou, J., Bi, H., Jiang, Q. and Gu, X. Heterogeneity analysis of astrocytes following spinal cord injury at single-cell resolution. GEO GSE189070. https://www.ncbi.nlm.nih.gov/geo/query/acc.cgi?acc=GSE189070
- Zhang, B., Zeng, K., Guan, R. and Yang, Y. Single-cell RNA-seq analysis reveals macrophage involvement in pathogenesis of human sporadic type A aortic dissection. GEO GSE213740. https://www.ncbi.nlm.nih.gov/geo/query/acc.cgi?acc=GSE213740
- Garcia-Bonilla, L., Racchumi, G. and Anrather, J. Brain and blood single-cell transcriptomic analysis in acute and subacute phases after experimental stroke. GEO GSE225948. https://www.ncbi.nlm.nih.gov/geo/query/acc.cgi?acc=GSE225948
- Zhang, Z., et al. A panoramic view of cell population dynamics in mammalian aging. GEO GSE247719. https://www.ncbi.nlm.nih.gov/geo/query/acc.cgi?acc=GSE247719 *Provides two data

samples

- Zhao, T., Sun, Z., Zhong, Q., Yu, X., Sun, T. and An, Z. Decoding SFRP2hi fibroblast progenitors in sustaining tooth growth in humans and mice at single-cell resolution. GEO GSE275119. https://www.ncbi.nlm.nih.gov/geo/query/acc.cgi?acc=GSE275119
- Bastidas-Ponce, A., et al. Comprehensive single-cell mRNA profiling reveals a detailed roadmap for pancreatic endocrinogenesis. GEO GSE132188. https://www.ncbi.nlm.nih.gov/geo/query/acc.cgi?acc=GSE132188
- Bandyopadhyay, S., Ahn, K. J., Duffy, M., Qin, L. and Tan, K. Mapping the cellular biogeography of human bone marrow niches using single-cell transcriptomics. GEO GSE253355. https://www.ncbi.nlm.nih.gov/geo/query/acc.cgi?acc=GSE253355
- Alkaslasi, M. R., et al. Single nucleus RNA-sequencing defines unexpected diversity of cholinergic neuron types in the adult mouse spinal cord. GEO GSE167597. https://www.ncbi.nlm.nih.gov/geo/query/acc.cgi?acc=GSE167597
- Wang, H., et al. Decoding human megakaryocyte development. GEO GSE144024. https://www.ncbi.nlm.nih.gov/geo/query/acc.cgi?acc=GSE144024
- Spildrejorde, M., et al. Gene expression analysis of hESCs undergoing neuronal differentiation [scRNA-seq]. GEO GSE192857. https://www.ncbi.nlm.nih.gov/geo/query/acc.cgi?acc=GSE192857
- Poscablo, D., Forsberg, C. and Medina, P. An age-specific platelet differentiation pathway from hematopoietic stem cells causes thrombocytosis and exacerbated thrombosis. GEO GSE255019. https://www.ncbi.nlm.nih.gov/geo/query/acc.cgi?acc=GSE255019
- Cosgrove, J. and Périe, L. Single-cell RNA sequencing of hematopoietic stem and progenitor cells from young and aged mice. GEO GSE226131. https://www.ncbi.nlm.nih.gov/geo/query/acc.cgi?acc=GSE226131
- Fast, E. External signals regulate continuous transcriptional states in hematopoietic stem cells. GEO GSE165844. https://www.ncbi.nlm.nih.gov/geo/query/acc.cgi?acc=GSE165844
- Simon, L. M. and Schiller, H. B. Longitudinal single-cell transcriptomics analysis of mouse lung upon bleomycin-induced injury. GEO GSE141259. https://www.ncbi.nlm.nih.gov/geo/query/acc.cgi?acc=GSE141259
- Linnarsson, S. Transcriptome analysis of single cells from the mouse dentate gyrus. GEO GSE95753. https://www.ncbi.nlm.nih.gov/geo/query/acc.cgi?acc=GSE95753
- Demerdash, Y., Bouman, B. J., Haghverdi, L. and Essers, M. A. Time series single-cell RNA sequencing of murine hematopoietic stem and progenitors (HSPCs) following in vivo IFNα treatment. GEO GSE226824. https://www.ncbi.nlm.nih.gov/geo/query/acc.cgi?acc=GSE226824
- Setty, M. Profiling of CD34+ cells from human bone marrow to understand hematopoiesis. ENA PRJEB37166. https://www.ebi.ac.uk/ena/browser/view/PRJEB37166
- Zhu, Y., Wang, T., Gu, J. and Pan, G. Characterization and generation of human definitive multipotent hematopoietic stem/progenitor cells. GEO GSE148215. https://www.ncbi.nlm.nih.gov/geo/query/acc.cgi?acc=GSE148215

- Dillon, L. Human bone marrow assessment by single-cell RNA sequencing, mass cytometry and flow cytometry. GEO GSE120446. https://www.ncbi.nlm.nih.gov/geo/query/acc.cgi?acc=GSE120446
- Teo, Y. V., Hinthorn, S. J., Webb, A. E. and Nicola, N. Single-cell RNA sequencing of aging peripheral blood. GEO GSE120505. https://www.ncbi.nlm.nih.gov/geo/query/acc.cgi?acc=GSE120505
- Paulson, K. G. and Chapuis, A. G. scRNA-seq reveals mechanisms of Merkel cell carcinoma acquired immunotherapy resistance [1]. GEO GSE117988. https://www.ncbi.nlm.nih.gov/geo/query/acc.cgi?acc=GSE117988 *Provides two data samples
- Sade-Feldman, M. and Yizhak, K. Defining T cell states associated with response to checkpoint immunotherapy in melanoma. GEO GSE120575. https://www.ncbi.nlm.nih.gov/geo/query/acc.cgi?acc=GSE120575
- Ghobrial, I. M. and Getz, G. Single-cell RNA sequencing reveals compromised immune microenvironment in precursor stages of multiple myeloma. GEO GSE124310. https://www.ncbi.nlm.nih.gov/geo/query/acc.cgi?acc=GSE124310
- Zhang, M., Yang, H., Liu, B. and Yan, X. Dissecting the transcriptomic landscape of human intrahepatic cholangiocarcinoma by single-cell RNA sequencing. GEO GSE138709. https://www.ncbi.nlm.nih.gov/geo/query/acc.cgi?acc=GSE138709
- Yanagawa, J., et al. Single-cell RNA sequencing of human early-stage lung adenocarcinoma with activating somatic KRAS mutations and associated normal lung tissues reveals alterations of key gene expression in alveolar type II pneumocytes. GEO GSE149655. https://www.ncbi.nlm.nih.gov/geo/query/acc.cgi?acc=GSE149655
- Jiang, H., Yu, D. and Yang, P. Transcriptional heterogeneity in primary and metastatic gastric cancer revealed using single-cell RNA sequencing. GEO GSE163558. https://www.ncbi.nlm.nih.gov/geo/query/acc.cgi?acc=GSE163558
- Lei, P., Pereira, E. R., Beyaz, S. and Padera, T. P. Single-cell sequencing reveals cancer cell heterogeneity in a murine breast cancer lymph node metastasis model. GEO GSE168181. https://www.ncbi.nlm.nih.gov/geo/query/acc.cgi?acc=GSE168181
- Jiang, T., Sun, S., Fan, Y. and Zhu, J. Spatiotemporal transcriptional atlas of lung adenocarcinoma from adenocarcinoma in situ to invasive carcinoma [scRNA-seq]. GEO GSE189357. https://www.ncbi.nlm.nih.gov/geo/query/acc.cgi?acc=GSE189357
- Wang, F. and Long, J. Single-cell and spatial transcriptome analysis reveals the cellular heterogeneity of liver metastatic colorectal cancer. GEO GSE225857. https://www.ncbi.nlm.nih.gov/geo/query/acc.cgi?acc=GSE225857
- Han, K., Joo, E. and Park, W. Single-cell RNA sequencing of primary breast cancer. GEO GSE228499. https://www.ncbi.nlm.nih.gov/geo/query/acc.cgi?acc=GSE228499
- Münter, D., et al. Multiomic analysis uncovers a continuous spectrum of differentiation and Wnt-MDK-driven immune evasion in hepatoblastoma [snRNA-seq]. GEO GSE283205. https://www.ncbi.nlm.nih.gov/geo/query/acc.cgi?acc=GSE283205

- Mehtonen, J., et al. Single-cell characterization of arrested B-lymphoid differentiation and leukemic cell states in ETV6-RUNX1-positive pediatric leukemia. GEO GSE148218. https://www.ncbi.nlm.nih.gov/geo/query/acc.cgi?acc=GSE148218
- Li, Z. and Luo, L. Single-cell RNA sequencing identifies molecular biomarkers predicting response to CDK4/6 inhibition in metastatic HR+/HER2- breast cancer. GEO GSE262288. https://www.ncbi.nlm.nih.gov/geo/query/acc.cgi?acc=GSE262288
- Geldhof, V., et al. Single-cell atlas identifies lipid-processing and immunomodulatory endothelial cells in healthy and malignant breast. GEO GSE155109. https://www.ncbi.nlm.nih.gov/geo/query/acc.cgi?acc=GSE155109 *Provides two data samples
- Yost, K. E., et al. Clonal replacement of tumor-specific T cells following PD-1 blockade [single cells]. GEO GSE123813. https://www.ncbi.nlm.nih.gov/geo/query/acc.cgi?acc=GSE123813 *Provides two data samples
- Wang, L., Dai, J., Han, R. and Jin, W. Single-cell map of diverse immune phenotypes in the metastatic brain tumor microenvironment of non-small cell lung cancer and triple-negative breast cancer. GEO GSE143423. https://www.ncbi.nlm.nih.gov/geo/query/acc.cgi?acc=GSE143423 *Provides two data samples
- Laughney, A. M., et al. The single-cell transcriptional landscape of human lung adenocarcinoma (primary tumors and metastases). GEO GSE123902. https://www.ncbi.nlm.nih.gov/geo/query/acc.cgi?acc=GSE123902
- Liu, Y., Ge, J., Chen, Y. and Yu, K. Single-cell profiling and spatial transcriptome of primary tumors and paired metastatic lymph nodes in breast cancer patients. GEO GSE225600. https://www.ncbi.nlm.nih.gov/geo/query/acc.cgi?acc=GSE225600
- Yang, J., Yu, J., Huang, X. and Li, Y. Single-cell network pharmacology predicts total therapies targeting multiple developmental clones in B-cell acute lymphoblastic leukemia. GEO GSE235787. https://www.ncbi.nlm.nih.gov/geo/query/acc.cgi?acc=GSE235787
- Chen, X. and Li, Z. Targeting tumor cells toward the antigenic specificity of bystander T cells in tumor microenvironment potentiates cancer immunotherapy. GEO GSE222002. https://www.ncbi.nlm.nih.gov/geo/query/acc.cgi?acc=GSE222002
- Zheng, C., et al. Landscape of infiltrating T cells in liver cancer revealed by single-cell sequencing. GEO GSE98638. https://www.ncbi.nlm.nih.gov/geo/query/acc.cgi?acc=GSE98638
- Cichocki, F. and Day, A. Nicotinamide enhances natural killer cell function and yields remissions in patients with non-Hodgkin lymphoma. GEO GSE222369. https://www.ncbi.nlm.nih.gov/geo/query/acc.cgi?acc=GSE222369
- Kumar, V., Ramnarayanan, K. and Tan, P. Single-cell atlas of lineage states, tumor microenvironment and subtype-specific expression programs in gastric cancer. GEO GSE183904. https://www.ncbi.nlm.nih.gov/geo/query/acc.cgi?acc=GSE183904
- Caron, M., et al. Single-cell analysis of childhood leukemia reveals a link between developmental states and ribosomal protein expression as a source of intra-individual heterogeneity. GEO

GSE132509. https://www.ncbi.nlm.nih.gov/geo/query/acc.cgi?acc=GSE132509

Furthermore, 82 single-cell ATAC sequencing datasets from the GEO database were included in this study.

- Morabito S, Miyoshi E, Swarup V. Single-nucleus chromatin accessibility and transcriptomic characterization of Alzheimer's Disease. GEO GSE174367. https://www.ncbi.nlm.nih.gov/geo/query/acc.cgi?acc=GSE174367
- Khateb M, et al. Transcriptomics, Regulatory Syntax, and Enhancer Identification in Heterogenous Populations of Mesoderm-Induced ESCs at Single-Cell Resolution. GEO GSE198730. https://www.ncbi.nlm.nih.gov/geo/query/acc.cgi?acc=GSE198730 *Provides two samples
- Wang G, Zhang D, Qin L, Huang B. Chemical-based external stimulation reprograms BJs into FiNs (scATAC-seq). GEO GSE206767. https://www.ncbi.nlm.nih.gov/geo/query/acc.cgi?acc=GSE206767
- Qiu X, Li Y, Brown M. Enhancer reactivation mediates adaptive resistance to FGFR inhibitors in triple-negative breast cancer [scATAC_seq]. GEO GSE168026. https://www.ncbi.nlm.nih.gov/geo/query/acc.cgi?acc=GSE168026
- Turner A, Shengen H, Zang C, Miller C. Cell-specific chromatin landscape in human coronary artery resolves regulatory mechanisms of disease risk. GEO GSE175621. https://www.ncbi.nlm.nih.gov/geo/query/acc.cgi?acc=GSE175621 *Provides four samples
- Wu LM, Lu QR. Transcriptional programs dictating Schwann cell transformation in MPNST [scATACseq]. GEO GSE178988. https://www.ncbi.nlm.nih.gov/geo/query/acc.cgi?acc=GSE178988 *Provides three samples
- Spildrejorde M, et al. Single-cell ATAC-seq analysis of hESCs undergoing neuronal differentiation [scATAC-seq]. GEO GSE192856. https://www.ncbi.nlm.nih.gov/geo/query/acc.cgi?acc=GSE192856 *Provides two samples
- Jimenez E, et al. A regulatory network of Sox and Six transcription factors initiate a cell fate transformation during hearing regeneration in adult zebrafish. GEO GSE192947. https://www.ncbi.nlm.nih.gov/geo/query/acc.cgi?acc=GSE192947 *Provides seven samples
- Giles J, Wherry EJ. Longitudinal single cell transcriptional and epigenetic mapping of effector, memory, and exhausted CD8 T cells reveals shared biological circuits across distinct cell fates [scATAC-Seq 1]. GEO GSE199556. https://www.ncbi.nlm.nih.gov/geo/query/acc.cgi?acc=GSE199556 *Provides two samples
- Li S, Yang M, Teng S, Wang D. Single-cell chromatin accessible state of paired primary and liver metastasis colorectal cancer cells from PDX cells derived mouse model. GEO GSE200813. https://www.ncbi.nlm.nih.gov/geo/query/acc.cgi?acc=GSE200813 *Provides six samples
- Li F, et al. Single cell ATAC-seq of mouse hair follicle morphogenesis. GEO GSE201213. https://www.ncbi.nlm.nih.gov/geo/query/acc.cgi?acc=GSE201213 *Provides three samples

- Lin YH, et al. Small intestine and colon tissue-resident memory CD8+ T cells exhibit transcriptional, epigenetic, and functional heterogeneity in concert with differential dependence on Eomesodermin [scATAC-seq]. GEO GSE205549. https://www.ncbi.nlm.nih.gov/geo/query/acc.cgi?acc=GSE205549 *Provides four samples
- Itahashi K, Irie T, Nishikawa H. Integrated multiomics profiling identifies the differentiation program of regulatory T cells in human tumors [scATAC-seq]. GEO GSE211087. https://www.ncbi.nlm.nih.gov/geo/query/acc.cgi?acc=GSE211087 *Provides eight samples
- Peter S, W Dean P. Temporal chromatin accessibility changes define transcriptional states essential for osteosarcoma metastasis [scATAC-Seq]. GEO GSE215758. https://www.ncbi.nlm.nih.gov/geo/query/acc.cgi?acc=GSE215758
- Spildrejorde M, et al. Single-cell ATAC-seq analysis of hESCs undergoing neuronal differentiation exposed to paracetamol [scATAC-seq]. GEO GSE220026. https://www.ncbi.nlm.nih.gov/geo/query/acc.cgi?acc=GSE220026 *Provides two samples
- Winter DR, Perlman H. Tissue-resident, extravascular Ly6c- monocytes are critical for inflammation in the synovium Figure 3 & 7. GEO GSE225801. https://www.ncbi.nlm.nih.gov/geo/query/acc.cgi?acc=GSE225801
- Liu Q, Dong W. scATAC-seq of antigen-specific CD8+ T cells from different time-course of LCMV-Armstrong infected mice. GEO GSE226067. https://www.ncbi.nlm.nih.gov/geo/query/acc.cgi?acc=GSE226067 *Provides five samples
- Chen R, et al. A multi-omics atlas of the human retina at single-cell resolution. GEO GSE226108. https://www.ncbi.nlm.nih.gov/geo/query/acc.cgi?acc=GSE226108 *Provides four samples
- Laurie SJ, et al. Multiomic Single Cell Evaluation Reveals Inflammatory Cytokines Affect Innate Lymphoid Cell Fate After Allogeneic Stem Cell Transplantation [scATAC-Seq]. GEO GSE232002. https://www.ncbi.nlm.nih.gov/geo/query/acc.cgi?acc=GSE232002 *Provides two samples
- Liu Q, Dong W. scATAC-seq of OT-1 CD8+ T cells from different time-course of Lm-OVA infected mice. GEO GSE241714. https://www.ncbi.nlm.nih.gov/geo/query/acc.cgi?acc=GSE241714 *Provides three samples
- Wu J, Castro LN, Suva ML, Bernstein BE. Single-cell transcription and chromatin accessbility profiles of IDH mutant gliomas. GEO GSE241745. https://www.ncbi.nlm.nih.gov/geo/query/acc.cgi?acc=GSE241745 *Provides seven samples
- Racine L, Parmentier R. Metabolic adaptation pilots the differentiation of human hematopoietic cells (scATAC-Seq). GEO GSE243004. https://www.ncbi.nlm.nih.gov/geo/query/acc.cgi?acc=GSE243004 *Provides four samples
- Chen R, et al. Single-cell multiomics of the human retina reveals hierarchical transcription factor collaboration in mediating cell type-specific effects of genetic variants on gene regulation. GEO GSE247157. https://www.ncbi.nlm.nih.gov/geo/query/acc.cgi?acc=GSE247157 *Provides six samples

- Poluben L, Balk SP, Russo J, Nouri M. Increased chromatin accessibility drives transition to androgen receptor splice variant dependence in castration-resistant prostate cancer [scATAC-Seq]. GEO GSE252843. https://www.ncbi.nlm.nih.gov/geo/query/acc.cgi?acc=GSE252843 *Provides three samples
- GSM8248668 - GSM8248676 *Provides nine samples
- Stomper J, et al. Sex differences in DNMT3A-mutant clonal hematopoiesis and the effects of estrogen [scATAC-Seq]. GEO GSE272265. https://www.ncbi.nlm.nih.gov/geo/query/acc.cgi?acc=GSE272265 *Provides two samples
- Baek S, et al. Single-cell multi-omics reveals tumor microenvironment factors underlying poor immunotherapy responses in ALK-positive lung cancer. GEO GSE274934. https://www.ncbi.nlm.nih.gov/geo/query/acc.cgi?acc=GSE274934 *Provides five samples
- Min Dai. An enhancer-AAV toolbox to target and manipulate distinct interneuron subtypes [P28]. GEO GSE277242. https://www.ncbi.nlm.nih.gov/geo/query/acc.cgi?acc=GSE277242
- Xu L, Korogi Y, Li R, Sun X. RUNX2 promotes pulmonary fibrosis through the conversion of alveolar fibroblasts into pathological fibroblasts. GEO GSE278419. https://www.ncbi.nlm.nih.gov/geo/query/acc.cgi?acc=GSE278419 *Provides two samples
- Kim D, Blackshaw S. Decoding Gene Networks Governing Hypothalamic and Prethalamic Neuron Development. GEO GSE284492. https://www.ncbi.nlm.nih.gov/geo/query/acc.cgi?acc=GSE284492 *Provides three samples
- Shen W. single-cell ATAC-sequencing of human aortic media that was composed by vascular smooth muscle cells. GEO GSE286575. https://www.ncbi.nlm.nih.gov/geo/query/acc.cgi?acc=GSE286575
- Roudier MP, et al. Patterns of intra- and inter-tumor phenotypic heterogeneity in men with lethal prostate cancer [scATAC-seq]. GEO GSE292194. https://www.ncbi.nlm.nih.gov/geo/query/acc.cgi?acc=GSE292194 *Provides three samples
- Cha P, et al. Integrative Single-Cell RNA and ATAC Sequencing Reveals the Impact of Chronic Cigarette Smoking on Lung Epithelial Responses to Influenza and Hyperoxia. GEO GSE294221. https://www.ncbi.nlm.nih.gov/geo/query/acc.cgi?acc=GSE294221 *Provides six samples
- Salem MA, et al. Gene regulatory programs of NK cells show that NCAM1 (CD56) and KIRs are controlled by genetically polymorphic distal regulatory elements [scATAC-seq]. GEO GSE297712. https://www.ncbi.nlm.nih.gov/geo/query/acc.cgi?acc=GSE297712
